# Cross-Architecture Knowledge Distillation for Histopathological Image Analysis

**DOI:** 10.1109/access.2026.3683880

**Published:** 2026-04-14

**Authors:** SEDDIK BOUDISSA, HIROHARU KAWANAKA, BRUCE ARONOW, V. B. SURYA PRASATH

**Affiliations:** 1 Graduate School of Engineering, Mie University, Mie 514-8507, Japan; 2 Division of Biomedical Informatics, Cincinnati Children’s Hospital Medical Center, Cincinnati, OH 45229, USA; 3 Department of Pediatrics, University of Cincinnati College of Medicine, Cincinnati, OH 45257, USA; 4 Department of Biomedical Informatics, College of Medicine, University of Cincinnati, Cincinnati, OH 45267, USA

**Keywords:** Histopathology, image classification, knowledge distillation, vision transformers, centered kernel alignment, kernel canonical correlation analysis

## Abstract

Histopathological image analysis presents unique challenges due to subtle inter-class variations, complex tissue structures, and high intra-class heterogeneity. Convolutional neural networks (CNNs) have traditionally dominated this domain by leveraging strong inductive biases toward locality, while vision transformers (ViTs) have recently emerged as a promising alternative due to their ability to model long-range dependencies. However, ViTs often struggle to capture fine-grained spatial patterns critical to histopathology, particularly when trained on limited data. In this work, we propose a knowledge distillation (KD) framework that transfers spatial and semantic knowledge from a CNN teacher to a ViT student while explicitly addressing representation and spatial misalignment between heterogeneous architectures. We analyze the limitations of conventional feature based distillation methods, which commonly rely on naive layer matching or pixel wise feature alignment, leading to suboptimal knowledge transfer. To overcome these issues, we introduce a principled layer alignment strategy based on representation similarity analysis using centered kernel alignment (CKA) and kernel canonical correlation analysis (KCCA). These measures enable architecture agnostic comparison of internal representations and facilitate the identification of semantically aligned stages between teacher and student models without enforcing strict spatial correspondence. Furthermore, we propose a stage-level representation alignment mechanism that preserves semantic consistency across representations while accommodating architectural differences between CNNs and ViTs, ensuring effective knowledge transfer at both representation and spatial levels. Extensive experiments on the BreakHis histopathological dataset demonstrate consistent performance improvements, particularly across fine-grained subtypes. Notably, our method achieves 96.87% using a ViT-Large student with a ResNet152 teacher on the patient-wise split setting and 98.82% accuracy on the image-wise subtype split, outperforming the best-performing state-of-the-art KD-based methods by 4% showing the promise of cross architecture KD in computational pathology.

## I. INTRODUCTION

Histopathological image analysis plays a critical role in cancer diagnosis and prognosis. Unlike natural images, histopathological images exhibit high microscopic variability, complex tissue organization, and subtle differences between classes, where discriminative cues often appear at fine spatial scales. Variations in staining, tissue morphology, and acquisition conditions further increase the difficulty of reliable automated analysis. These properties make representation learning for histopathology fundamentally challenging, and demand models that can capture both local structural patterns and global contextual relationships.

Convolutional neural networks (CNNs) [1], [2], [3], [4], [5] have traditionally been the predominant models for histopathological image analysis due to their strong inductive bias [6] toward locality and translation invariance. Their hierarchical structure enables effective modeling of low-level textures and cellular patterns. More recently, vision transformers (ViTs) [7] have emerged as a powerful alternative, demonstrating strong performance across a wide range of vision tasks [8], [9], [10], [11], [12]. By relying on self-attention mechanisms, ViTs are capable of modeling long-range dependencies and global interactions, which are particularly appealing for complex tissue structures where contextual information is essential. Several studies have explored the application of ViTs to medical and histopathological imaging, reporting promising results. However, ViTs lack the built-in locality bias of CNNs and often require large-scale data [13], [14], [15] to generalize effectively. In histopathology, where annotated data is limited and variations are subtle, this limitation becomes critical. A natural direction to address this issue is to transfer the inductive knowledge of CNNs into ViTs, combining the locality-awareness of convolutional models with the expressive global modeling of attention-based architectures.

A widely adopted framework for transferring knowledge between models is knowledge distillation (KD) [16], [17], [18], [19]. In response-based distillation, the student model learns from the soft predictions of a teacher model, providing class-level guidance. Feature-based distillation, on the other hand, aligns internal representations between teacher and student models, offering richer supervision at intermediate layers. While feature-based distillation is particularly appealing for transferring structural and semantic information, it introduces significant challenges when applied to architecturally heterogeneous models, such as CNNs and ViTs. One major challenge in feature-based distillation [20], [21], [22], [23], [24] is layer and spatial misalignment. CNNs and ViTs differ substantially in depth, stage structure, and feature map resolution. Naively matching corresponding layers or enforcing pixel-to-pixel alignment between feature maps is often suboptimal and may even hinder learning. Some existing approaches attempt strict spatial alignment by resizing or interpolating feature maps, or by enforcing one-to-one correspondence at the pixel level. However, such strategies overlook the fact that representations may be semantically aligned without being spatially aligned, especially across fundamentally different architectures.

To address layer alignment, one could perform a full pairwise comparison between all layers of the teacher and student models. While comprehensive, this approach quickly becomes computationally expensive and difficult to interpret, especially for deep networks. Moreover, matching based purely on feature dimensions or spatial resolution does not guarantee semantic consistency. To overcome these limitations, we leverage centered kernel alignment (CKA), a representation similarity metric designed to compare neural network representations based on the geometry of their feature spaces. Rather than enforcing direct feature or spatial correspondence, CKA measures how similarly two layers organize data samples relative to each other. Importantly, CKA is architecture-agnostic, invariant to isotropic scaling and orthogonal transformations, and well-suited for comparing representations across heterogeneous models. These properties make it particularly attractive for guiding layer alignment in feature-based KD. However, while CKA effectively captures linear representational similarity, it may fail to reveal deeper nonlinear semantic relationships between feature spaces. This limitation becomes especially relevant in histopathological settings, where class distinctions are often nonlinear and subtle. To fill this gap, we incorporate kernel canonical correlation analysis (KCCA), which extends CCA into a reproducing kernel Hilbert space. By modeling nonlinear dependencies between representations, on top of CKA, KCCA provides a complementary measure that captures nonlinear dependencies between representations.

By jointly leveraging CKA and KCCA, our proposed approach ensures that teacher-student layer alignment is driven by semantic similarity rather than architectural coincidence, while avoiding rigid spatial constraints. Furthermore, we introduce a strategy to align stage level representations in a structure aware manner, accommodating differences in feature map resolution and architectural design between CNNs and ViTs. This allows effective knowledge transfer without enforcing suboptimal pixel-level correspondence.

The main contributions of this work can be summarized as follows:

- A clear and unified background on representation similarity and feature-based KD, with intuitive explanations of existing methods.
- A novel layer alignment strategy for CNN to ViT distillation that combines CKA and KCCA “to capture both linear and nonlinear semantic similarities”, as well as a stage-level representation alignment strategy.
- Extensive experimental validation on the BreakHis dataset [25], including fine grained subtype classification across eight histopathological classes, demonstrating the effectiveness of the proposed approach.

The remainder of this paper is organized as follows. In Section II, we review related work on KD, representation similarity measures, and CNN-Transformer hybrid approaches, with a focus on feature based alignment strategies. In Section III, we present our proposed method in detail, including the theoretical foundations of CKA and KCCA and their integration into the distillation framework. Section IV reports extensive experimental results on the BreakHis histopathological dataset, including evaluations on patient wise and image-wise splits, along with implementation details and ablation studies.

## II. BACKGROUND

We provide a concise background on the challenges associated with histopathological image analysis and review representative knowledge distillation (KD) approaches proposed in the literature. We focus on widely adopted KD paradigms, including both response based and feature based distillation methods, and summarize the core intuition underlying each approach. To enhance clarity and comparability, the reviewed methods illustrative diagrams are provided to highlight their key mechanisms. Rather than exhaustively detailing implementation specifics, our objective is to distill the fundamental ideas behind these approaches and to identify their inherent limitations. We believe that several of these limitations are effectively addressed by the proposed method.

### A. HISTOPATHOLOGICAL IMAGE ANALYSIS

**FIGURE 1.**
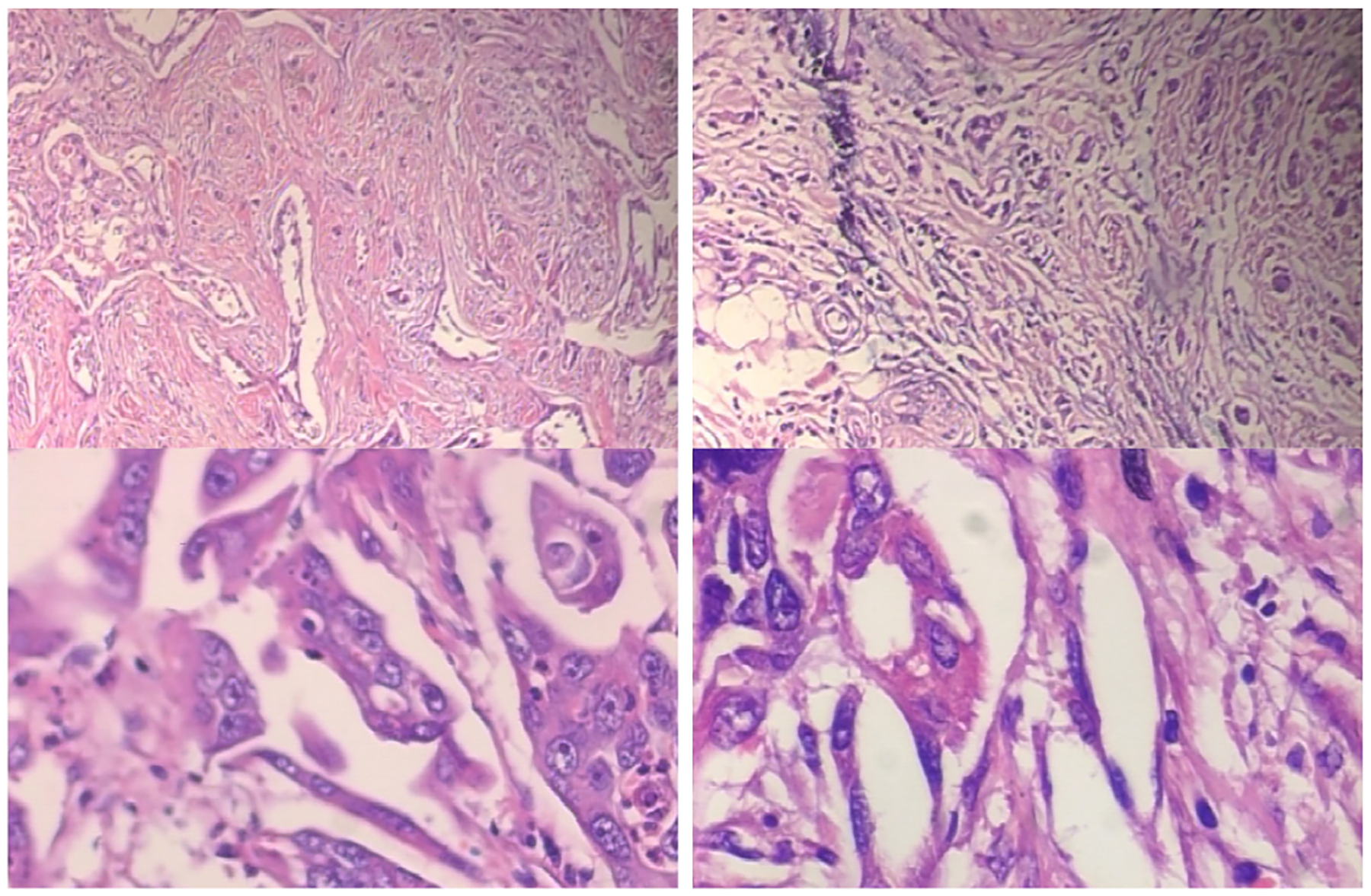
Example WSIs (Malignant Ductal Carcinoma) exhibiting multiple scales from the BreakHis dataset [25] - 40x, 100x, 200x, and 400x.

Histopathological images are microscopic visual representations of tissue samples, typically obtained through biopsy or surgical resection and stained using protocols such as hematoxylin and eosin (H & E). These images play a critical role in disease diagnosis, grading, and prognosis, particularly in oncology, where subtle cellular and structural patterns carry significant clinical meaning. Despite their importance, histopathological image analysis presents several challenges. First, whole-slide images (WSIs) are extremely large, often reaching gigapixel resolutions, which makes direct processing computationally prohibitive. As a result, WSIs are commonly divided into smaller patches, introducing challenges related to context loss and patch aggregation. Second, the microscopic nature of histopathology leads to high intra class variability and low inter-class separability, requiring models to capture fine grained texture, morphology, and spatial organization across multiple scales Figure 1. Third, annotated data is often limited, as pixel or region level labeling requires expert pathologists and is time-consuming, costly, and prone to inter-observer variability. To address these constraints, multiple instance learning (MIL) has been widely adopted in histopathological analysis. In MIL-based frameworks, a WSI is treated as a bag of instances (patches), where slide-level labels are available but instance-level annotations are absent. This formulation enables models to learn discriminative patterns from weak supervision while reducing annotation burden, making MIL particularly suitable for large-scale histopathological datasets.

### B. DISTILLING THE KNOWLEDGE IN A NEURAL NETWORK

Hinton et al [26] introduced KD as a framework for transferring the knowledge of a large teacher model into a compact student model optimized for deployment. The core idea is that soft class probabilities produced by the teacher encode inter-class similarity information that is absent in hard labels, enabling improved student generalization. Training with soft targets reduces the gradient variance compared to one-hot supervision, leading to smoother optimization. However, standard softmax outputs often suppress informative low-probability classes. To address this, a temperature parameter T is introduced to soften the output distribution,

(1) $$q_{i}=\frac{\text{exp}\left(z_{i}/T\right)}{\sum_{j}\text{exp}\left(z_{j}/T\right)}.$$

As T increases, the softened distribution reveals richer class relationships. Logit matching [27] can be viewed as a special case of this formulation at high temperatures. The student is trained using a weighted combination of soft-target distillation loss and hard-label supervision,

(2) $${ℒ}_{\text{KD}}=\alpha T^{2}\;\text{KL}\left(p_{t}^{\left(T\right)}\|p_{s}^{\left(T\right)}\right)+(1-\alpha )\text{CE}(y,p_{s}),$$

where α balances the two objectives and the $T^{2}$ term compensates for temperature-scaled gradients.

### C. FitNets

**FIGURE 2.**
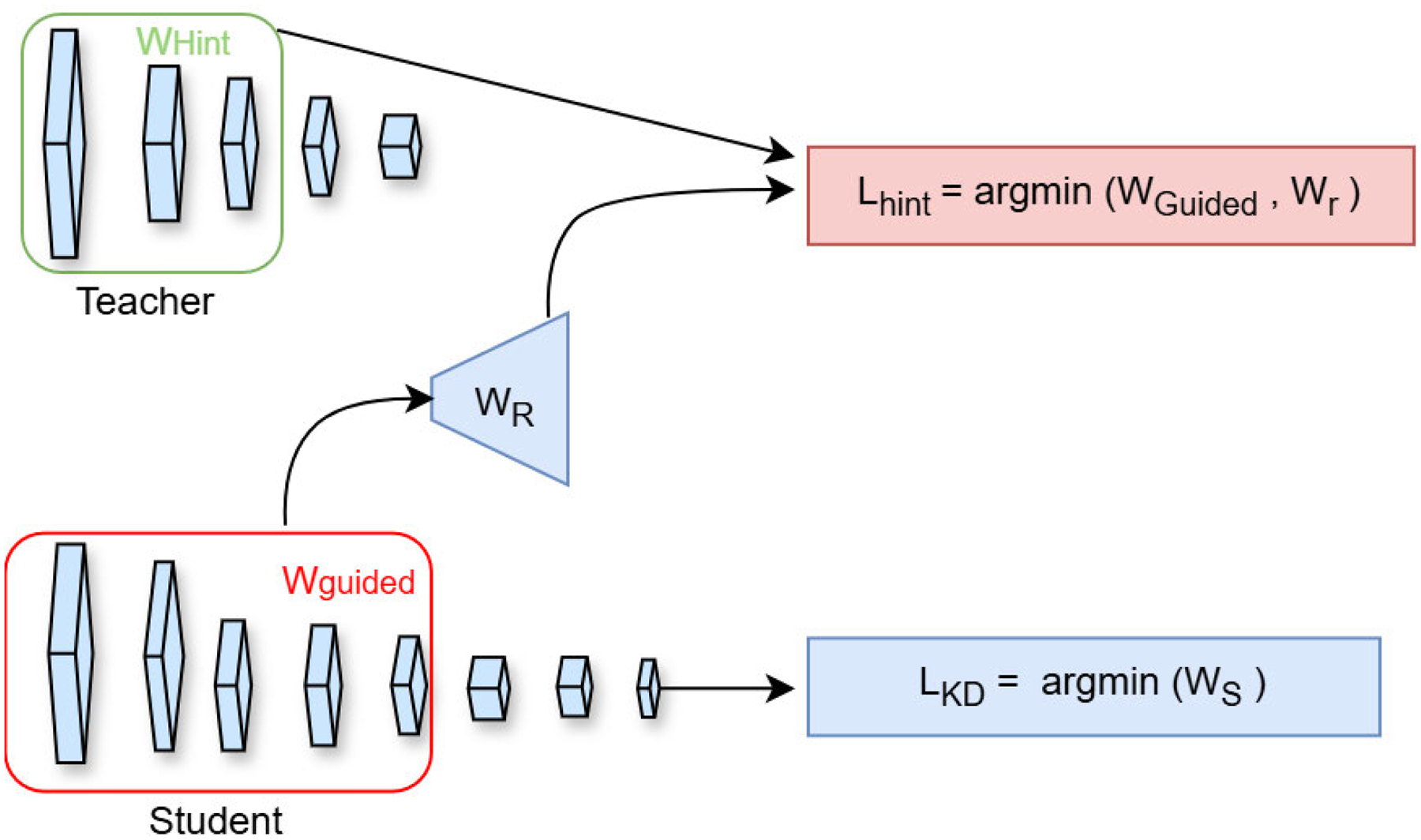
FitNet -Training a deeper student network using mid-layer hints and output signals.

Beyond output based distillation methods that match softened class distributions [26], [28], FitNets [29] introduce intermediate feature supervision to train deeper yet significantly thinner student networks. Motivated by theoretical and empirical evidence that network depth improves representational capacity and generalization [30], [31], [32], [33], FitNets guide the student using mid level teacher feature maps, referred to as hints. To address architectural mismatches between teacher and student, a lightweight regressor is applied to the student’s guided layer to align spatial and channel dimensions. The intermediate supervision is imposed through a hint-based regression loss,

(3) $${ℒ}_{HT}\left(W_{\text{Guided}},W_{r}\right)\;=\frac{1}{2}{\left\|u_{h}\left(x;W_{\text{Hint}}\right)-r\left(v_{g}\left(x;W_{\text{Guided}}\right);W_{r}\right)\right\|}^{2},$$

where $u_{h}$ and $v_{g}$ denote the teacher and student subnetworks up to the hint and guided layers, respectively, and r is the regressor parameterized by $W_{r}$. Importantly, the hint loss is applied at carefully selected mid-layers rather than deep layers, reducing semantic mismatch and easing optimization when training very deep students. After this hint-based pretraining stage, the student is further optimized using standard distillation with soft and hard targets following [26], see Figure 2. Extensive experiments demonstrate that FitNets can outperform larger teachers while using substantially fewer parameters and lower computational cost.

### D. CUMULATIVE SPATIAL KD (CSKD)

**FIGURE 3.**
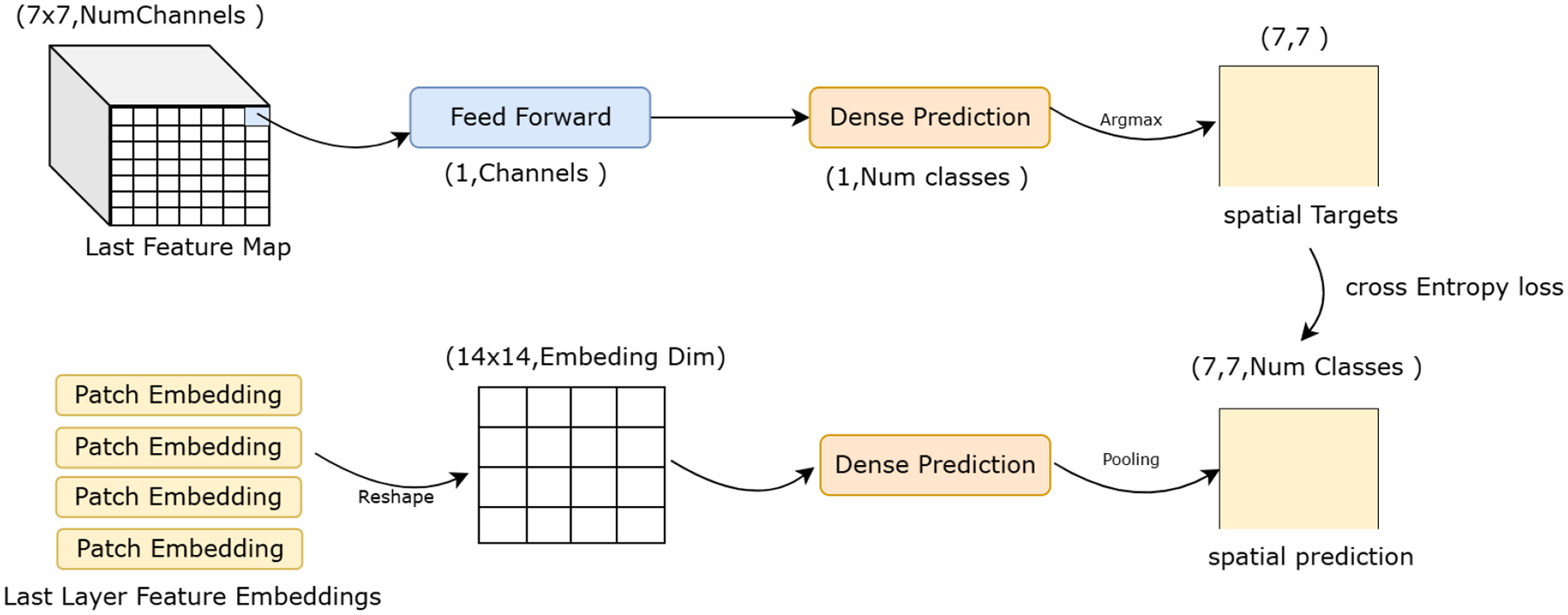
CSKD - Dense predictions feature maps alignment between teacher and student.

CSKD [21] addresses the challenges of distilling knowledge from a CNN teacher to a ViT student by avoiding direct feature alignment and instead transferring spatial-wise knowledge through dense predictions. The method is motivated by two key issues: the semantic mismatch between CNN and ViT intermediate features, which renders conventional feature mimicking ineffective, and the risk of suppressing the ViT’s global modeling capability due to the strong local inductive bias of CNNs. To overcome these limitations, CSKD introduces dense prediction distillation, where class distributions are generated at each spatial location from the final feature maps of both networks. The CNN teacher produces dense class predictions over its final spatial feature map, while the ViT student generates patch-wise predictions that are spatially pooled to match the teacher’s resolution, enabling element-wise supervision via cross-entropy loss. To further balance local and global knowledge transfer, CSKD incorporates a Cumulative Knowledge Fusion (CKF) module that dynamically adjusts the contribution of spatial (local) and global supervision signals during training, progressively emphasizing global knowledge to preserve the ViT’s global representation capacity, see Figure 3. The overall objective combines dense spatial supervision and global distillation losses, weighted adaptively through the CKF mechanism,

(4) $${ℒ}_{\text{TOT}}=\frac{1}{2}{ℒ}_{\text{CE}}+\frac{1}{2}{ℒ}_{\text{distill}}+{ℒ}_{\text{CSKD}}.$$

### E. CROSS-LAYER DISTILLATION WITH SEMANTIC CALIBRATION

Traditional feature map based KD methods typically rely on manually predefined teacher-student layer pairs to transfer intermediate representations [34], [35], [36], [37], [38]. Two common strategies are widely adopted in the literature: one pair matching, where a single manually selected teacher student layer pair is used for distillation, and one to one matching, where layers of the same depth are aligned up to min(Ls,Lt), the minimum number of layers between the student and teacher networks. However, layers at comparable depths across heterogeneous architectures often encode different semantic concepts. Such semantic mismatch can introduce negative regularization, forcing the student to imitate teacher representations that are not semantically aligned, ultimately degrading generalization performance.

To address this limitation, the SemCKD framework [39] introduces Semantic Calibration, a principled mechanism for automatically learning cross-layer associations. Instead of fixing layer correspondences, SemCKD considers the complete set of possible teacher student layer pairs,

(5) $$𝒞=\left\{\left(s_{l},t_{l}\right)|\forall \;s_{l}\in \left[1,\ldots ,s_{L}\right],\;t_{l}\in \left[1,\ldots ,t_{L}\right]\right\},$$

and employs an attention-based module to learn soft, instance specific association weights between each student layer and all teacher layers. For a given input instance i, the semantic association weight is computed as,

(6) $$\alpha_{\left(s_{t},t_{t}\right)}[i]=ℱ\left(F_{s}^{s}[i],F_{t}^{t}[i]\right),$$

where F() denotes the learned attention function operating on student and teacher feature representations.

To ensure compatibility between heterogeneous architectures, student feature maps are first projected via a lightweight convolutional transformation to match the spatial dimensions of the corresponding teacher layers:

(7) $$F_{t_{l}}^{s^{\prime }}=\text{Proj}\left(F_{s_{l}}^{s}\in R^{b\times c_{s}\times h_{s_{l}}\times w_{s_{l}}},\;t_{l}\right),\;t_{l}\in \left\{1,\ldots ,T_{L}\right\}.$$

Semantic similarity between layers is then estimated by constructing pairwise similarity matrices using dot product relationships among samples within each layer, capturing the intrinsic relational structure of the representations. These similarity matrices are embedded into query key representations to produce a soft alignment distribution across teacher layers. The final cross-layer distillation loss is,

(8) $${ℒ}_{\text{CLD}}=\sum_{s_{l}=1}^{S_{L}}\sum_{t_{l}=1}^{T_{L}}\alpha \left(s_{l},t_{l}\right)[i]\text{MSE}\left(F_{t}^{t_{l}}[i],F_{s}^{s_{l}}[i]\right),$$

where the attention weights modulate the contribution of each teacher-student layer pair.

Importantly, SemCKD work [39] establish a theoretical connection between the learned attention weights and the Orthogonal Procrustes problem, showing that higher attention corresponds to lower orthogonal transformation error between teacher and student representations. This result provides a geometric interpretation of the attention mechanism as an indicator of semantic alignment. Despite its effectiveness, SemCKD presents several limitations. First, semantic similarity is inferred primarily through feature-space correlations, which may not fully capture deeper semantic equivalence, especially when spatial positions or channel dimensions encode different meanings across architectures. Second, the exhaustive comparison across all teacher student layer pairs introduces notable computational overhead, even though pooling operations are applied to mitigate feature map dimensionality.

### F. DATA-EFFICIENT EARLY KD FOR VISION TRANSFORMERS (DearKD)

DearKD [40] aims to improve the data efficiency of transformers through feature based and response-based KD, using a two-stage framework. In the first stage, guidance signals are provided to the student, and in the second stage, the transformer is allowed to train freely to avoid being suppressed by the teacher. In the DearKD framework, the authors do not explicitly provide a one-to-one mapping between specific layers of the CNN teacher and the ViT student; they only mention that knowledge is transferred from early intermediate layers. In contrast, the framework addresses the issue of spatial alignment by using an aligner, which transforms a set of tokens at a certain layer to match the dimensions of the corresponding CNN feature map.

#### 1) STAGE I

In Stage I of DearKD, the goal is to inject convolutional inductive biases from a CNN teacher into the early layers of a ViT student. The teacher provides intermediate feature maps rich in local patterns like edges and textures which serve as fixed supervision targets. To enable meaningful comparison, the student’s patch tokens are passed through an aligner module that reshapes and transforms them into a spatial format compatible with the teacher’s convolutional features. This aligner consists of a reshape operation, bilinear interpolation to match spatial resolution, followed by depth-wise convolution to introduce local filtering, and then LayerNorm and ReLU to normalize the features. The aligned student representation is then compared to the teacher’s feature map using a mean squared error (MSE) loss, allowing the student to absorb spatial priors without altering the teacher’s outputs. This distillation is confined to Stage I, after which the transformer trains independently, free to develop its own global representations. DearKD enhances this process by embedding relative positional encoding (RPE) into the transformer’s attention mechanism. Unlike absolute positional encoding, which assigns fixed positions to tokens, RPE allows the model to learn relative spatial relationships between patches. This makes the attention more sensitive to local structure, effectively mimicking convolutional behavior without sacrificing the flexibility of self attention. The loss of dearKD Stage I is as follows,

(9) $$L=\alpha L_{\text{CE}}+(1-\alpha )L_{\text{logit}}+\beta L_{\text{hidden}}.$$

#### 2) STAGE II

In Stage II of DearKD, the student Vision Transformer continues training independently, without further guidance from the CNN teacher. The distillation signal from Stage I is removed to avoid suppressing the ViT’s capacity for global representation learning. However, the relative positional encoding introduced earlier is retained, allowing the self-attention mechanism to simulate convolution-like behavior by learning spatial relationships between patches. During this stage, the ViT is trained using standard cross-entropy loss on the class token output, with no additional distillation loss applied. This design ensures that while the student has absorbed local inductive biases in Stage I, it is free to develop its own global semantic structure in Stage II. The authors of [40] emphasize that this two-stage framework improves data efficiency and avoids over-regularization, allowing the transformer to balance local and global feature learning. The loss of DearKD Stage II is as follows,

(10) $$L=L_{\text{CE}}(\text{logit},y).$$

### G. KD VIA THE TARGET-AWARE TRANSFORMER

The token alignment transformer (TAT) [41] addresses the issue of spatial misalignment between teacher and student feature maps. Specifically, it handles the problem of one-to-one (pixel-to-pixel) distillation commonly used in CNN-based models, which assumes that the corresponding spatial positions in the teacher and student feature maps carry the same semantic meaning. However, due to architectural differences, such as varying receptive fields and feature extraction strategies, the semantic correspondence between features at the same spatial position often does not hold.

Previous one-to-one distillation methods typically transfer knowledge from the 2D representations of the 3D flattened feature maps, where each spatial position in the teacher is directly compared to the same position in the student, under the assumption that both maps share identical height (H) and width (W). This rigid alignment can lead to suboptimal knowledge transfer when the two models have different spatial semantics,

(11) $$f^{s^{⊤}}=\left[f_{1}^{s},f_{2}^{s},f_{3}^{s},\ldots ,f_{N}^{s}\right],$$

(12) $$f^{t^{⊤}}=\left[f_{1}^{t},f_{2}^{t},f_{3}^{t},\ldots ,f_{N}^{t}\right].$$

where $f_{i}^{s}$ and $f_{i}^{t}$ stands for the 2d flattened 3d positions from the teacher and the student respectively. The feature matching approach is given by,

(13) $${ℒ}_{FM}={\left\|F^{S}-F^{T}\right\|}_{2}=\sum_{i=1}^{N}{\left\|f_{i}^{s}-f_{i}^{t}\right\|}_{2}.$$

**FIGURE 4.**
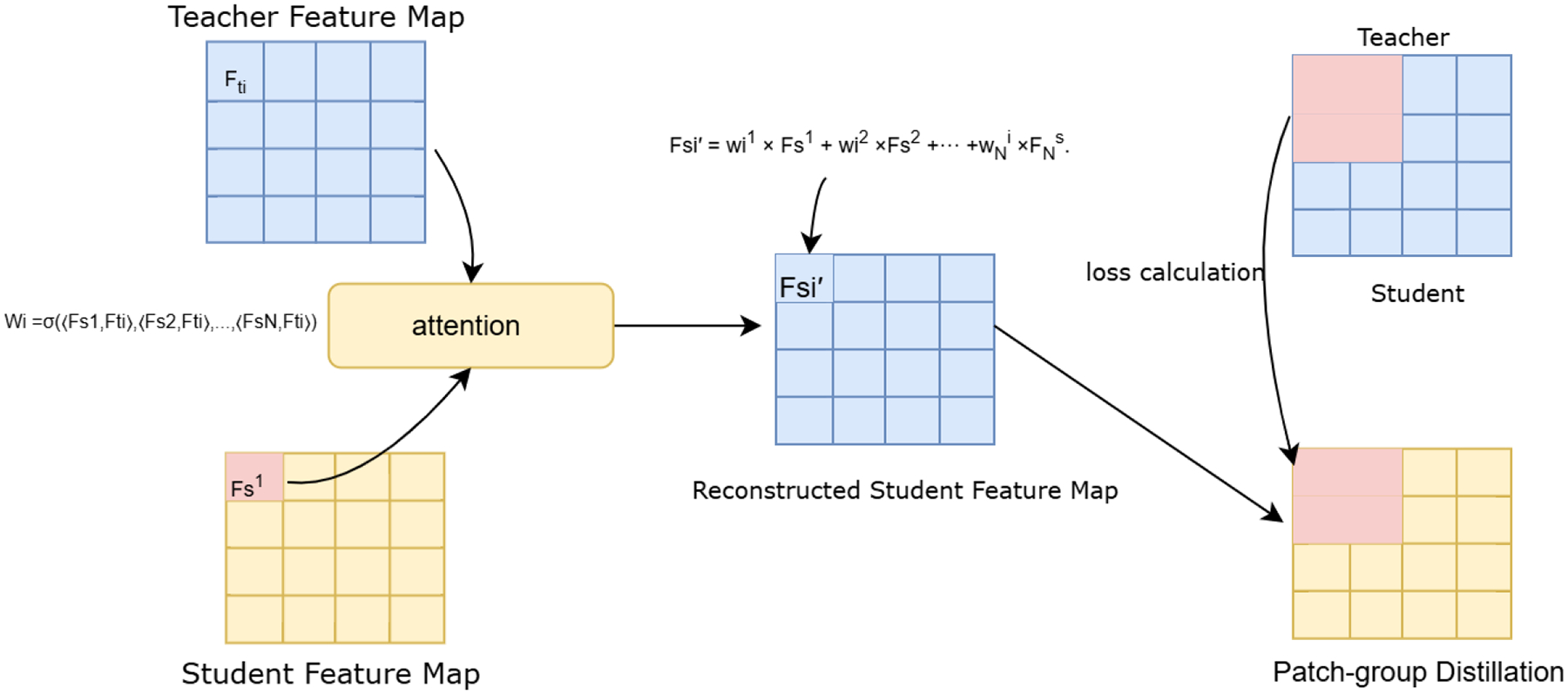
TAT - KD via the target-aware transformer.

The authors of TAT [41] proposed a one-to-all distillation approach, where each location in the student’s feature map receives supervisory signals from all spatial locations of the teacher’s feature map, weighted by their similarity. To achieve this, TAT rebuilds every position (or pixel) in the student feature map by aggregating information from all other locations in the student feature map, guided by the similarity between the corresponding teacher position and each student position, computed through attention similarity. For example, the position $F_{1}^{s}$ in the student feature map is reconstructed using all other student positions $F_{i}^{s}$ after being compared with the corresponding teacher position $F_{1}^{t}$, as illustrated in Figure 4.

After reconstructing the spatially-aware student feature map, the distillation loss is computed as the summation of distances between the corresponding source (teacher) and target (student) features at each spatial location. For computational efficiency, TAT restricts the distillation process to local patch groups within the feature maps. The feature map is divided into smaller patches, and both the reconstruction of spatially-aware student features and the distillation process are performed within these localized regions. Complementing the local patch-group distillation, anchor-point distillation captures global semantics. Here, each feature map is pooled (e.g., through average pooling) into anchor vectors, forming compact global representations. These anchor features are then used for another layer of distillation through TAT.

### H. CENTERED KERNEL ALIGNMENT (CKA)

The centered kernel alignment (CKA) [42], [43], [44], [45] is computed as,

(14) $$\text{CKA}=\frac{\frac{1}{k}\sum_{i=1}^{k}A\left(X_{i}X_{i}^{⊤},Y_{i}Y_{i}^{⊤}\right)}{\sqrt{A\left(X_{i}X_{i}^{⊤},X_{i}X_{i}^{⊤}\right)}+\sqrt{A\left(Y_{i}Y_{i}^{⊤},Y_{i}Y_{i}^{⊤}\right)}},$$

where we denote A(⋅,⋅) the Hilbert-Schmidt independence criterion (HSIC),

$$\text{A}\left(K,L\right)=\frac{1}{n\left(n-3\right)}\left(\text{tr}\left(\tilde{K}\tilde{L}\right)+\frac{1^{⊤}\tilde{K}11^{⊤}\tilde{L}1}{\left(n-1\right)\left(n-2\right)}-\frac{2}{n-2}1^{⊤}\tilde{K}\tilde{L}1\right).$$

HSIC measures the similarity of activations between two layers or networks by comparing their pairwise relationships across a batch of inputs. Importantly, HSIC can be computed even when the two layers have different feature dimensions, since it operates on kernel (Gram) matrices $K=XX^{⊤}$ and $L=YY^{⊤}$, both of which reduce to an n×n similarity structure, where n is the batch size. However, raw HSIC values are scale-dependent and unbounded, making them difficult to interpret or compare across layers. CKA addresses this by normalizing HSIC, ensuring that the similarity values lie between 0 and 1 and are invariant to isotropic scaling of the representations. This normalization makes it possible to meaningfully compare representation similarities across different architectures or layers, even when the activation vectors have different shapes.In equation (15), we have K and L, which are defined as $K=XX^{⊤}$ and $L=YY^{⊤}$, respectively. Here, X denotes the activations (representations) of a given layer for all images in a batch. The product $XX^{⊤}$ quantifies the pairwise similarity between the input samples according to the representation learned at layer $L_{1}$. Similarly, $YY^{⊤}$ captures the similarity between the same inputs as represented by layer $L_{2}$. The matrix X has size (p,n), where p is the representation dimension and n is the batch size.

HSIC Equation (15) consists mainly of three terms. The first term, $\text{tr}(\tilde{K}\tilde{L})$ is the trace of the product of the centered Gram matrices $\tilde{K}$ and $\tilde{L}$. Here, $\tilde{K}$ and $\tilde{L}$ are obtained from $K=XX^{⊤}$ and $L=YY^{⊤}$, respectively, after centering to remove self-similarities (diagonal elements). This term captures the alignment between the similarity structures of the two layers. The middle and last terms in the unbiased HSIC estimator serve as correction factors that eliminate bias caused by finite sample sizes. The middle term captures the product of global similarities within each kernel matrix, acting as a baseline for what similarity one would expect by chance. The last term adjusts for overlap between the two kernels, ensuring that the final HSIC score reflects true dependence rather than inflated alignment. Together, these terms stabilize the estimator, making it robust to batch size and enabling meaningful comparison across layers or models.

Intuitively, if the relative similarities between inputs are preserved across layers, the CKA (or HSIC) value will be high, indicating that the layers learn similar representations. Conversely, if these relative similarities differ, the layers are learning different aspects of the data. Although CKA is effective for measuring representation similarity, a high CKA score does not necessarily imply high semantic similarity between layers. CKA captures geometric relations between instances within a representation, whereas semantic similarity refers to layers encoding comparable types of features. This limitation is particularly relevant in heterogeneous settings (e.g., CNN-ViT), where layers may share similar relational structure while representing different semantics. These observations motivate the use of complementary alignment methods that explicitly learn correlated subspaces across teacher and student representations, such as kernel canonical correlation analysis (KCCA).

### I. KERNEL CANONICAL CORRELATION ANALYSIS (KCCA)

Principal component analysis (PCA) is a classical unsupervised method for analyzing and reducing the dimensionality of a single dataset. Given high-dimensional observations, PCA identifies a set of orthogonal directions (principal components) defined as linear combinations of the original features. These components are ordered by the amount of variance they explain, with the first component capturing the maximum variance and each subsequent component capturing the largest remaining variance under orthogonality constraints. While PCA is effective for summarizing and compressing a single representation, it operates in a single-view setting and does not account for relationships between multiple feature spaces. In practical scenarios, the same samples are often represented through different models or architectures, such as CNNs and Vision Transformers (ViTs). Applying PCA independently to each representation reveals dominant variance directions within each space, but provides no guarantee that the resulting components are aligned or comparable across views. As a result, PCA cannot capture shared or correlated structure between different representations.

Canonical correlation analysis (CCA) addresses this limitation by explicitly modeling the relationship between two paired datasets. Given two feature matrices corresponding to the same set of samples, CCA seeks linear projections for each view such that the projected variables are maximally correlated. Unlike PCA, which maximizes variance within a single representation, CCA maximizes cross-view correlation, producing pairs of canonical variables that define a shared latent space. The associated eigenvalues reflect the strength of correlation between views rather than explained variance. Consequently, CCA provides a principled framework for comparing and aligning representations extracted by different models processing the same inputs. However, CCA assumes that the relationship between the two views can be adequately described by linear transformations. This assumption is often restrictive for deep neural representations, which are shaped by highly nonlinear operations and may originate from architectures with fundamentally different inductive biases. As a result, linear CCA may fail to capture complex dependencies between heterogeneous deep feature spaces.

Kernel canonical correlation analysis (KCCA) [46] extends CCA by leveraging the kernel trick to model nonlinear relationships between views [47]. By implicitly mapping each representation into a high-dimensional reproducing kernel Hilbert space, KCCA performs CCA in this kernel-induced space, enabling the discovery of nonlinear correlations without explicitly computing the mappings. This makes KCCA particularly well suited for aligning and comparing deep neural representations across models or layers, where linear assumptions are insufficient.

## III. METHOD

### A. KNOWLEDGE DISTILLATION

Knowledge distillation (KD) [26], [27], [28], [29] is a technique originally proposed to compress a large or complex model or an ensemble of models into a smaller student network. In response-based distillation, the student learns from the teacher’s output distribution, typically the soft probabilities produced by the teacher or the averaged outputs of an ensemble. Through this process, the student can capture the dark knowledge contained in the teacher’s predictions, especially the informative structure in the incorrect-class probabilities, which reflect how the teacher generalizes across the data. In our work, the objective of distillation is different. Rather than compressing the entire knowledge of the teacher, our goal is to transfer only selected aspects of the teacher’s behavior that are beneficial for guiding the student.

One of the well-known challenges in response-based KD is the over-suppression of the student. When the teacher’s signal is too strong, the student is forced to mimic the teacher too closely, which can drag the student away from learning its own complementary representations and may lead to overfitting the teacher’s biases. Therefore, a crucial design point in our approach is defining how much teacher signal should be transferred, and at which stage of training. For feature-based KD [23], [34], [36], knowledge is typically transferred from an intermediate layer of the teacher to a corresponding intermediate layer of the student. The goal is to provide mid-level representational guidance so that the student can benefit from the teacher’s learned hierarchical features. However, directly forcing the student’s feature map at position “i” to match the teacher’s feature at the same position often referred to as layer-to-layer distillation [21] implicitly assumes that both layers encode information of the same semantic level. In practice, this assumption does not hold. Teacher and student networks may differ substantially in architecture, depth, receptive fields, and feature extraction mechanisms. Consequently, their corresponding layers can represent different levels of semantic abstraction. When there are differences like this, strict layer to layer matching can misguide the student The student may be pushed to replicate high-level or domain-specific representations that it is not yet capable of forming. Conversely, the teachers signal may impose low-level patterns that suppress the student’s ability to learn its own useful representations. These issues can result in overfitting (the student memorizes teacher features beyond its representational capacity) or underfitting (the student cannot reproduce complex teacher information and effectively ignores it). A further challenge is spatial misalignment, which arises both in homogeneous architectures (CNN to CNN) and, more severely, in heterogeneous settings such as CNN-to-ViT distillation - our case. Even when architectures are similar, small variations in feature resolution, stride, or receptive field can hinder meaningful alignment of feature maps. In heterogeneous architectures, the discrepancy becomes fundamental; CNNs represent visual information as dense grid-structured feature maps, whereas Vision Transformers operate on flattened token sequences enriched with positional embeddings. This structural gap makes it difficult to enforce direct feature consistency.

### B. CKA AND KCCA FOR LAYERS ALIGNMENT

To overcome the issues mentioned earlier, we employ a general solution based on centered kernel alignment (CKA) and kernel canonical correlation analysis (KCCA) [46]. CKA [42] is a powerful similarity metric that compares the representational structure of layers from different models. As explained in Section II, CKA quantifies how similar two layers are by focusing not on raw activations, but on the geometric relationships between samples. In other words, it measures the similarity of instance relationships across layers, providing a robust and architecture agnostic way to identify layers that preserve spatial dimensionality. Moreover, A useful identity in representational similarity analysis connects example-level similarity structures to feature-level interaction patterns. Let $X\in R^{n\times d_{x}}$ and $Y\in R^{n\times d_{y}}$ denote the representations of the same n examples in two layers. The following equality holds,

(15) $$\left\langle \text{vec}\left(XX^{\text{T}}\right),\text{vec}\left(YY^{\text{T}}\right)\right\rangle =\text{tr}\left(XX^{\text{T}}YY^{\text{T}}\right)={\left\|Y^{\text{T}}X\right\|}_{F}^{2}.$$

The left-hand side, $\text{vec}{\left(XX^{⊤}\right)}^{⊤}\text{vec}\left(YY^{⊤}\right)$, measures the similarity between the inter-example similarity matrices of the two layers. The matrices $\left(XX^{⊤}\right)$ and $\left(YY^{⊤}\right)$ encode dot-product similarities between examples in each representation space; a high value therefore indicates that the two layers organize the dataset’s examples in a similar geometric structure. the same quantity can be written in terms of feature-level interactions, as shown on the right-hand side. The term ${\left\|Y^{⊤}X\right\|}_{F}^{2}$ sums the squared dot products between every feature in X and every feature in Y, and is is large when the subspaces spanned by the features of X and Y overlap strongly even if individual features do not co-activate or correspond one-to-one.

However, it is important to recognize a key limitation of CKA when it is used to compare internal layers. Although CKA provides a robust measure of how similarly two layers organize examples, a high CKA score does not imply that the layers encode the same semantic information. Instead, CKA primarily reflects whether the two representations induce similar geometric relationships among examples, regardless of the specific feature detectors that produce these responses. CKA can yield high similarity scores even between layers that belong to different abstraction stages particularly in architectures such as Transformers, where many layers share similar relational structure. This occurs because CKA operates by comparing inter instance relations it constructs Gram matrices $K\in R^{n\times n},\;K_{ij}=\text{Gram}\left(x_{i},\;x_{j}\right),\;L\in R^{n\times n},\;L_{ij}=\text{Gram}\left(y_{i},y_{j}\right)$ that capture example-example similarities in each representation, and then measures the alignment between these relational structures. Two layers may therefore appear highly similar if they produce comparable instance-level geometry, even when they rely on entirely different feature sets to distinguish between classes. Consequently, CKA alone cannot guarantee semantic alignment between layers and should not be solely relied upon for selecting corresponding teacher-student layers in KD.

To complement the geometry based similarity we use Kernel Canonical Correlation Analysis (KCCA) to detect shared (possibly nonlinear) factors between two layer representations. Canonical Correlation Analysis (CCA) seeks linear projections u and v that maximize the correlation between the projected views Xu and Yv. In complex, nonlinear setting classical CCA can fail to reveal meaningful shared structure because it is restricted to linear projections, KCCA overcomes this limitation by applying the kernel trick where each view is implicitly mapped into a reproducing kernel Hilbert space (RKHS) using a nonlinear feature map ϕ(), and CCA is then performed on this high dimensional space using Gram matrices This allows KCCA to capture nonlinear statistical dependencies between representations that may be invisible to geometry based measures like CKA.

In practice, KCCA follows a procedure analogous to classical CCA after mapping the data into kernel-induced feature spaces. First, the representations X and Y are mean-centered by subtracting the empirical mean of each feature to remove first-order statistics. Next, covariance and cross-covariance operators between the centered representations are computed in the kernel space using the corresponding Gram matrices. Canonical directions are then obtained by solving a generalized eigenvalue problem, which can be expressed via singular value decomposition (SVD) of the regularized cross-covariance operator. The resulting eigenvalues correspond to the canonical correlations, whose square roots quantify the strength of the shared components between the two views, while the associated eigenvectors define the projection directions that maximize correlation in the kernel space.

Let $X\in R^{n\times d_{x}}$ and $Y\in R^{n\times d_{y}}$ be the responses of two layers for the same n examples. we Choose kernels $k_{X}(\cdot ,\cdot )$ and $k_{Y}(\cdot ,\cdot )$ and form the centered Gram matrices $K_{X}$ and $K_{Y}$ (centered by $H=I_{n}-\frac{1}{n}11^{⊤}$),

$$K_{X}=H\;K_{X}^{\text{raw}}H,\;K_{X}^{\text{raw}}[i,j]=k_{X}\left(x_{i},x_{j}\right),$$

and similarly for $K_{Y}$. KCCA then finds coefficient vectors $\alpha ,\beta \in R^{n}$ that solve the generalized eigenproblem corresponding to maximizing the correlation of $\alpha^{⊤}K_{X}$ and $\beta^{⊤}K_{Y}$,

$$\underset{\alpha ,\beta }{\text{max}}\frac{\alpha^{⊤}K_{X}K_{Y}\beta }{\sqrt{\alpha^{⊤}\left(K_{X}+\kappa I\right)K_{X}\alpha }\sqrt{\beta^{⊤}\left(K_{Y}+\kappa I\right)K_{Y}\beta }},$$

where κ>0 is a small ridge (regularization) term added for numerical stability. Solving this yields canonical correlations $\rho_{1}\geq \rho_{2}\geq \ldots$.

We summarize the KCCA alignment of two layers by the mean of the top-m canonical correlations,

$$\overset{‾}{\rho }=\frac{1}{m}\sum_{j=1}^{m}\rho_{j}.$$

In our experiments, we instantiate $k_{X}$ and $k_{Y}$ as RBF kernels, enabling KCCA to capture nonlinear correspondences between teacher and student representations. A high KCCA score indicates that there exist nonlinear functions of the two representations that are strongly correlated across examples in other words, the two layers share common latent factors. In practice this suggests the layers encode similar informational content or abstraction axes, and therefore are better candidates for teacher student matching in distillation. Importantly KCCA is not a proof of identical semantics, but it provides stronger evidence of shared, potentially nonlinear structure than linear measures or geometric-only metrics such as CKA.

### C. TEACHER STUDENT LAYERS ALIGNMENT

Previous studies have shown that CKA often yields block structured similarity matrices across depth, even when comparing different architectures such as CNNs, ViTs, and Swin Transformers. These blocks indicate that large segments of one model’s hierarchy correspond to differently positioned segments in another model. For example, the first 60 layers of one model may align with the first 40 layers of another, suggesting that both cover a comparable representational scope despite architectural differences.

Leveraging this observation, we begin by computing a CKA similarity matrices between CNN and ViT layers. To reduce computation while retaining global representational meaning, we use the CLS token for ViT layers and global average pooled (GAP) features for CNN layers. The CLS token is specifically trained to summarize all patch embeddings, making it suitable as a compact global descriptor.

Both the teacher and student networks are divided into three stages (early, mid, and late) by uniformly partitioning their layers into equal thirds based on depth. Then we select, for each student layer, a set of candidate teacher layers with the highest CKA values within the corresponding stage. However, while CKA measures geometric similarity between example relations, it does not guarantee that two layers encode the same semantic abstractions. To address this limitation, we refine each CKA selected pair using Kernel CCA (KCCA), again computed on CLS/GAP features. KCCA identifies maximally correlated nonlinear shared subspaces between two feature sets, providing a more semantically meaningful alignment than CKA alone. For each candidate teacher layer Ti and student layer Sj we compute the mean of the top m canonical correlations,

$$\overset{‾}{\rho }\left(T_{i},S_{j}\right)=\frac{1}{m}\sum_{k=1}^{m}\rho_{k}.$$

**FIGURE 5.**
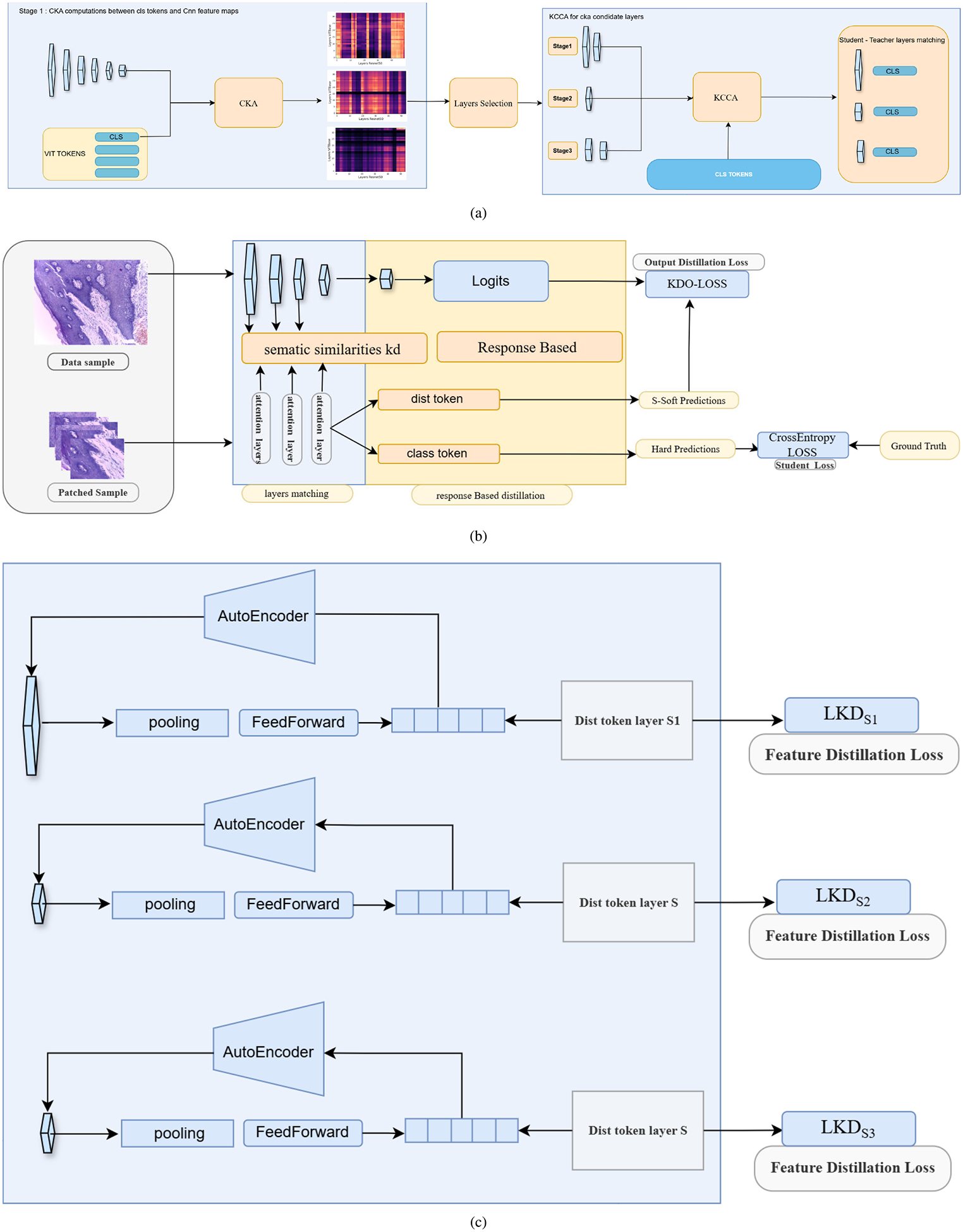
Overall methodology illustrating (a) teacher student layers alignment, (b) response based distillation, and (c) teacher student feature maps alignment.

We then choose the final matched teacher-student layers as,

$$ST_{j}^{*}=\text{arg}\underset{T_{i},S_{i}\in {𝒞}_{j}}{\text{max}}\;\overset{‾}{\rho }\left(T_{i},S_{j}\right),$$

where Cj denotes the set of CKA-selected candidates for student layer Sj. This combined CKA, KCCA pipeline ensures that knowledge transfer occurs between layers that share both geometric similarities and nonlinear semantic alignment. It avoids the common misinterpretation of using CKA alone for layer matching and ultimately promotes more meaningful, abstraction aware distillation between heterogeneous architectures, see Figure 5.

### D. TEACHER STUDENT FEATURE MAPS STAGE-LEVEL REPRESENTATION ALIGNMENT AND RESPONSE BASED DISTILLATION

KD from CNNs to ViTs is challenging due to fundamental architectural and representational differences. CNNs rely on local inductive biases and produce hierarchical spatial feature maps through convolutional operations, whereas Vision Transformers process non overlapping patch embeddings and employ self attention to model global and long range dependencies. Consequently, layers that are semantically related in the teacher and student do not necessarily encode spatially aligned information, making direct feature matching across corresponding spatial locations ineffective. To address this mismatch, we extend response based distillation by introducing three learnable distillation tokens, each associated with a specific stage of the Vision Transformer, namely early, intermediate, and late layers. These tokens are introduced following the same mechanism as the distillation token in data-efficient image transformers (DeiT) [48] and are treated analogously to the classification token within the transformer architecture. Each distillation token aggregates information from its corresponding student layer and serves as an interface for receiving teacher supervision at a specific level of abstraction.

**FIGURE 6.**
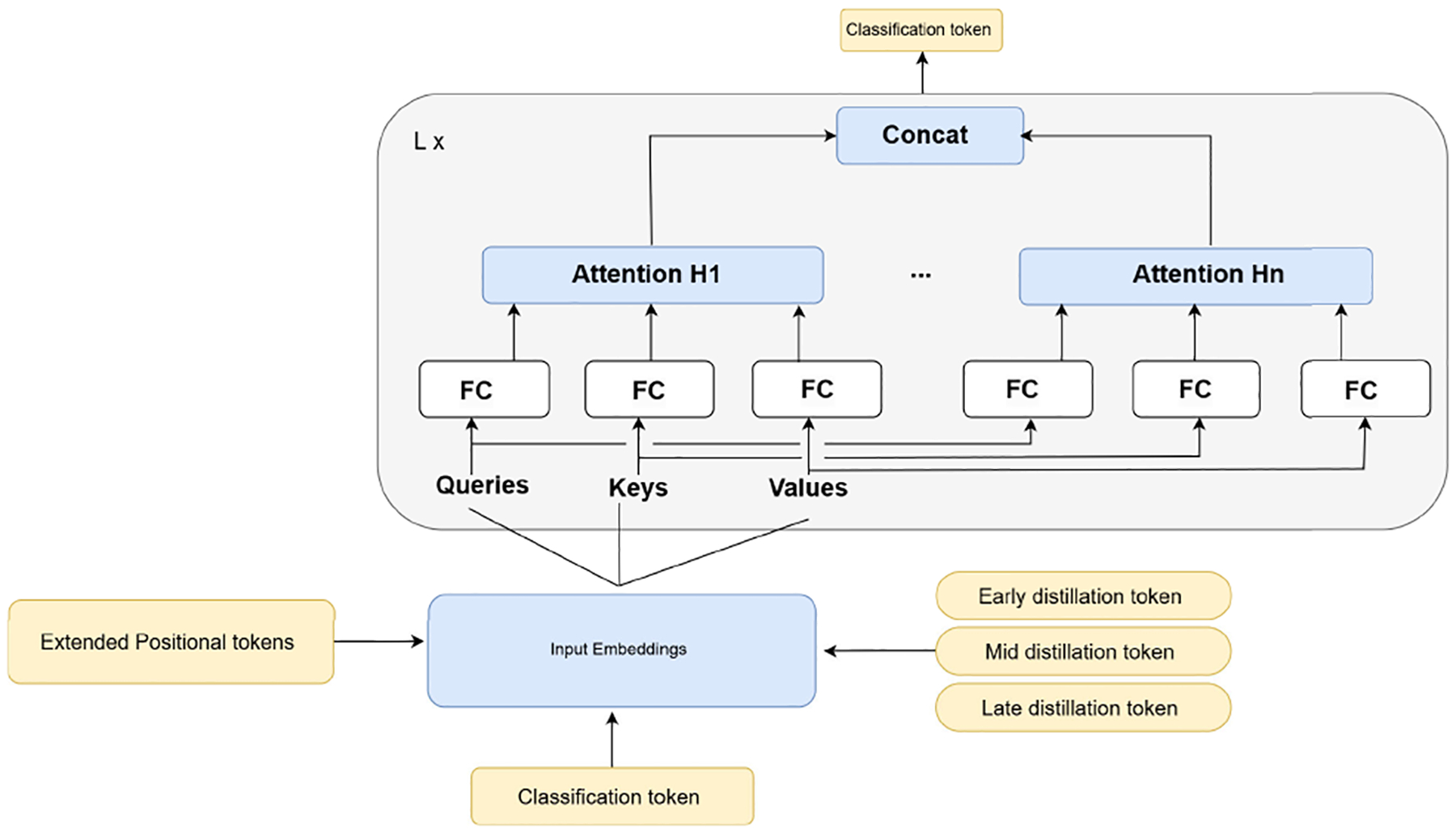
Proposed token augmentation: three distillation tokens are appended to the input sequence and propagated through all transformer layers.

The proposed tokens are propagated through all transformer encoder layers without interruption. At predefined depths (early, mid, late), we extract the hidden representations of the corresponding distillation tokens to compute the loss with the corresponding teacher’s layer. After extraction, the tokens continue to flow through subsequent layers, ensuring that the overall computation graph remains unchanged, Figure 6. This design preserves the standard architecture, with the only modification being an increased token sequence length. As a result, pretrained weights can be directly reused without modification, except for a straightforward extension of the positional embeddings to account for the additional tokens. This ensures full compatibility with standard pretrained ViT models while enabling effective multi stage feature distillation. This design enables the student to selectively absorb knowledge from different depths of the teacher network while preserving flexibility in its internal representations. In addition, it allows an explicit analysis of which abstraction stages contribute most effectively to student learning.

A key challenge in intermediate feature distillation is spatial misalignment, since CNN representations preserve spatial structure while Vision Transformer tokens are global vectors. To bridge this gap, we apply spatial pooling followed by a learned MLP projection to the teacher feature maps, transforming them into compact vectors that match the dimensionality of the student distillation tokens. Each distillation token is trained to align with its corresponding projected teacher representation, forming a dedicated and stage specific knowledge transfer pathway. Formally, let $F_{l}^{T}$ denote the teacher feature map at layer 1. The projected teacher representation is computed as,

(16) $$v_{l}^{T}=\text{MLP}\left(\text{Pool}\left(F_{l}^{T}\right)\right)\in R^{D},$$

where Pool denotes global average pooling and D is the dimensionality of the student distillation token. Instead of enforcing strict Euclidean matching, we employ a cosine similarity based loss to align the projected teacher vector $v_{l}^{T}$ with the corresponding student distillation token $t_{l}^{S}$,

(17) $${ℒ}_{\text{feat,}l}=1-\frac{\left\langle v_{l}^{T},t_{l}^{S}\right\rangle }{{\left\|v_{l}^{T}\right\|}_{2}{\left\|t_{l}^{S}\right\|}_{2}}.$$

This objective encourages directional agreement between representations while remaining invariant to scale, which is particularly suitable for aligning heterogeneous feature spaces.

To further improve the quality of the projected teacher representations, the teacher side MLP is trained in an unsupervised encoder decoder manner to reconstruct the original feature map. This auxiliary objective encourages the projected vectors to preserve informative and structured characteristics of the teacher features throughout training. The total feature distillation loss, combining early, mid, and late stages, is defined as,

(18) $$L_{\text{feat}}=\alpha_{f}L_{\text{feat},\;\text{early}}+\beta_{f}L_{\text{feat},\;\text{mid}}+\gamma_{f}L_{\text{feat},\;\text{late}},$$

where $\alpha_{f},\beta_{f},\gamma_{f}$ control the relative contribution of each abstraction stage.

We defined binary weights over three bridge stages (early, middle, late), indicating whether feature-level distillation is applied at each stage. We evaluate three configurations:

- **(1, 1, 0):** Distillation is applied to early and middle stages, while late-stage alignment is disabled. This is our primary setting. It is motivated by the observation that early and intermediate representations are more transferable across architectures, whereas late CNN features remain spatially localized, in contrast to the global attention based representations in ViTs.
- **(0, 1, 1):** Distillation is applied to middle and late stages, while early layers are left unconstrained. This configuration serves as a comparative setting to evaluate the effect of removing low-level guidance.
- **(1, 1, 1):** Distillation is applied across all stages, corresponding to full feature alignment and serving as a baseline.

This design avoids extensive hyperparameter tuning and instead provides a controlled analysis of how stage-wise alignment affects cross-architecture knowledge transfer.

In addition to feature-based supervision, we incorporate standard response-based distillation on the output logits. The response-based loss is defined as,

(19) $$L_{KD}=KL\left(\sigma \left(\frac{z_{t}}{T}\right)\|\sigma \left(\frac{z_{s}}{T}\right)\right)\cdot T^{2},$$

where $z_{t}z_{s}$ and are the teacher and student logits, T is the temperature, and σ(⋅) denotes the softmax function. Finally, the overall training objective is given by a weighted sum of the classification, response-based, and feature-based distillation losses,

(20) $$L_{\text{total}}=\alpha L_{\text{cls}}+\left(1-\alpha \right)L_{\text{kd}}+\gamma L_{\text{feat}},$$

where α,β,γ are hyperparameters controlling the relative importance of each supervision signal.

### E. AUTOENCODER PROJECTOR

Following the layer matching phase, we instantiate three independent autoencoder (AE) projectors, one for each selected teacher–student bridge pair. Each AE is dynamically sized according to the feature map dimensions of its corresponding teacher layer. Each AE consists of an encoder and a symmetric decoder, both implemented as two-layer MLPs. Given a teacher feature map $F\in R^{C\times H\times W}$, the encoder first applies adaptive average pooling to obtain a channel-wise representation $f\in R^{C}$. This vector is then projected through a linear layer to a hidden dimension of 512, followed by LayerNorm and GELU activation, and finally mapped to a latent vector $z\in R^{192}$, matching the embedding dimension of the student tokens. The decoder mirrors this process by mapping z back to a vector of dimension C, reconstructing the pooled representation $\overset{ˆ}{f}$. Since spatial information is removed by the pooling operation, the autoencoder operates on channel-wise statistics rather than attempting to reconstruct the original spatial feature map. The AE projectors are trained jointly with the distillation process without any pre-training stage. Specifically, their parameters are optimized end-to-end using a reconstruction loss between f and $\overset{ˆ}{f}$, together with the feature alignment objective. This encourages the latent representation z to preserve informative channel-level characteristics that facilitate effective teacher–student alignment.

## IV. EXPERIMENTAL RESULTS AND DISCUSSION

### A. SETUP, DATASETS, AND METRICS

During the distillation phase, both the teacher and student models are trained jointly throughout all stages. The teacher is initialized with pretrained ImageNet weights and fine-tuned on the target dataset alongside the student. For layer matching, within each of the three predefined stages (early, middle, late), we first compute all pairwise CKA similarities between candidate teacher and student layers. The top 3 layer pairs per stage are retained based on CKA scores. KCCA is then applied to these candidates, and the single best pair is selected for each stage resulting in a total of three bridge pairs. For KCCA, we use an RBF kernel, a regularization term is applied with $\kappa =1\times 10^{-4}$. The KCCA similarity is computed as the mean of the top-m canonical correlations, with m=10. Both CKA and KCCA are computed once after the warm-up phase and remain fixed during the subsequent distillation process.

**FIGURE 7.**
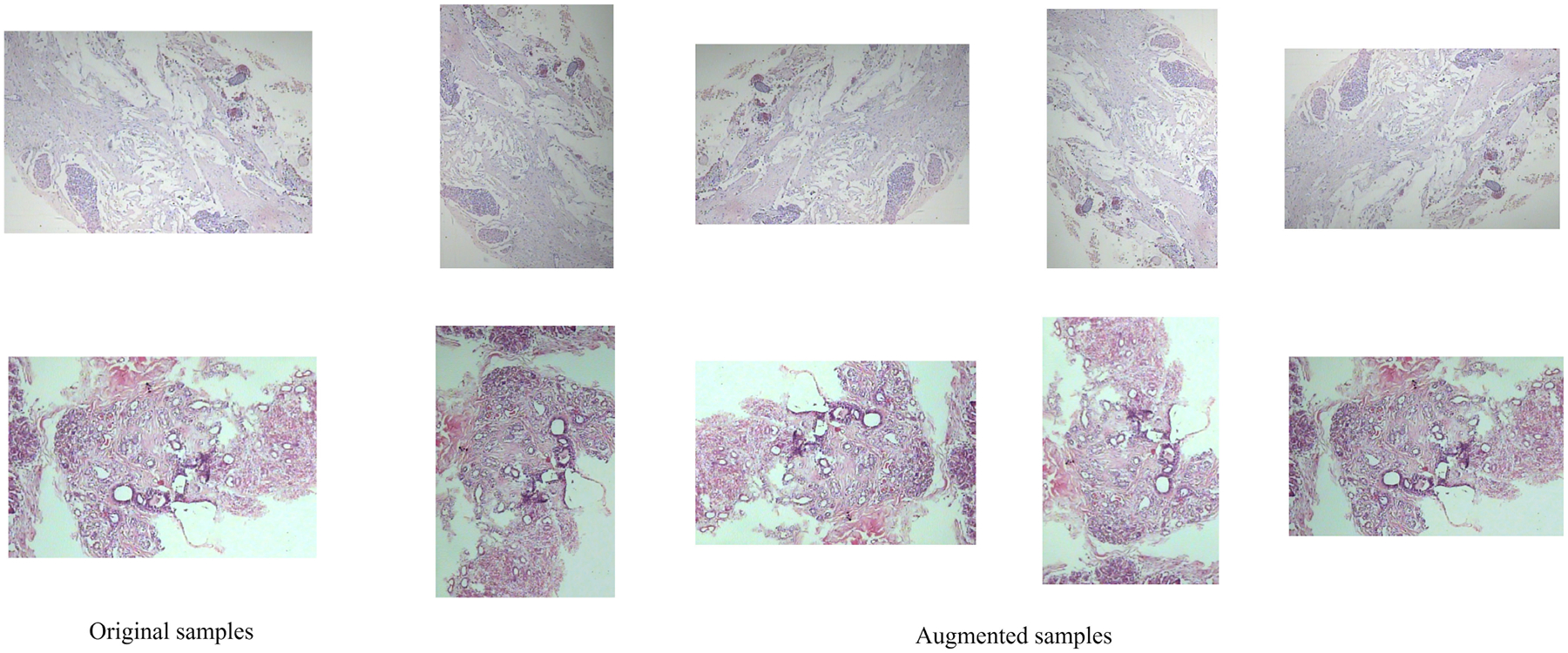
Data augmentation examples.

We evaluate our method on the widely used BreakHis [25] dataset (Table 1) which contains 7,909 Hematoxylin and Eosin (H&E) breast histopathology images from 82 patients at four magnifications (40×, 100×, 200×, 400×). Images are labeled as benign or malignant, with four histological subtypes in each group. The benign subclasses contain adenosis, fibroadenoma, phyllodes tumor, tubular adenoma; and malignant subclasses contain ductal, lobular, mucinous, papillary (Table 2). We report multiclass (8 subtypes) results for both patient-wise and image wise splits. This dataset has been extensively used in developing and testing AI models for histopathological image analysis, and prior studies have exclusively utilized this popular dataset due to its controlled acquisition settings and availability of clear metadata at the patient level. The image-wise evaluation protocol remains widely adopted in prior works on the BreakHis dataset, enabling direct and fair comparison with existing methods. While patient-wise evaluation provides a more reliable estimate of generalization, reporting image-wise results is still valuable for benchmarking consistency within the literature. In our experiments, the BreakHis dataset is divided into 60% for training, 20% for validation, and 20% for testing. This split has been consistently applied across all experiments. ensuring a balanced representation of classes across all subsets. During training, all images were resized to 224 × 224 pixels, converted to tensors, and normalized using the standard ImageNet mean and standard deviation (mean = [0.485, 0.456, 0.406], std = [0.229, 0.224, 0.225]). For the BreakHis dataset, we applied four data augmentation techniques. Examples of augmented samples are shown in Figure 7. The applied augmentations (rotation, flips, color jitter, and random blur) were deliberately mild, as medical images are highly sensitive to aggressive transformations that may distort diagnostically relevant structures.

We tested multiple base models including cnns used as teachers in our framework, ResNet50 [1], ResNet152, VGG19 [32], AlexNet [1], DenseNet [53], EfficientNet [54], along with different variants of and attention-based models refs for the student network - ViT-Base [7], ViT-Large, Swin-Small [55] and their corresponding details are given in Table 3. All models are trained using the SGD optimizer with a batch size of 16, a weight decay of 0.001, and for 50 epochs. For some model configurations, we found that employing a learning rate scheduler was beneficial, enabling smaller and more refined updates in the later stages of training. Specifically, a step scheduler with a step size of 5 and a decay factor (γ) of 0.1 was used. All experiments were conducted on an NVIDIA GeForce RTX 3090 GPU with 24 GB of GDDR6X memory, using CUDA version 12.4. To evaluate the models, we used the following evaluation metrics:

- Accuracy: The correctness of the model’s predictions across all classes. accuracy is calculated as the sum of all correct predictions overall predictions.
- Precision: This measures the accuracy of positive predictions. It is the ratio of true positive predictions (correctly predicted positive instances) to the total predicted positive instances (both true positives and false positives). Precision focuses on minimizing false positives.
- Recall: This measures the ability of a model to identify positive instances correctly. It is the ratio of true positive predictions to the total actual positive instances (true positives and false negatives). Recall focuses on minimizing false negatives. An important parameter to consider in medical imaging classification tasks is the false negative predictions which refers to the number of instances where the truth is positive whereas the model prediction is negative.

### B. MAGNIFICATION-INDEPENDENT MULTI-CATEGORY (MIM) CLASSIFICATIONS-IMAGE-WISE AND PATIENT-WISE RESULTS

**FIGURE 8.**
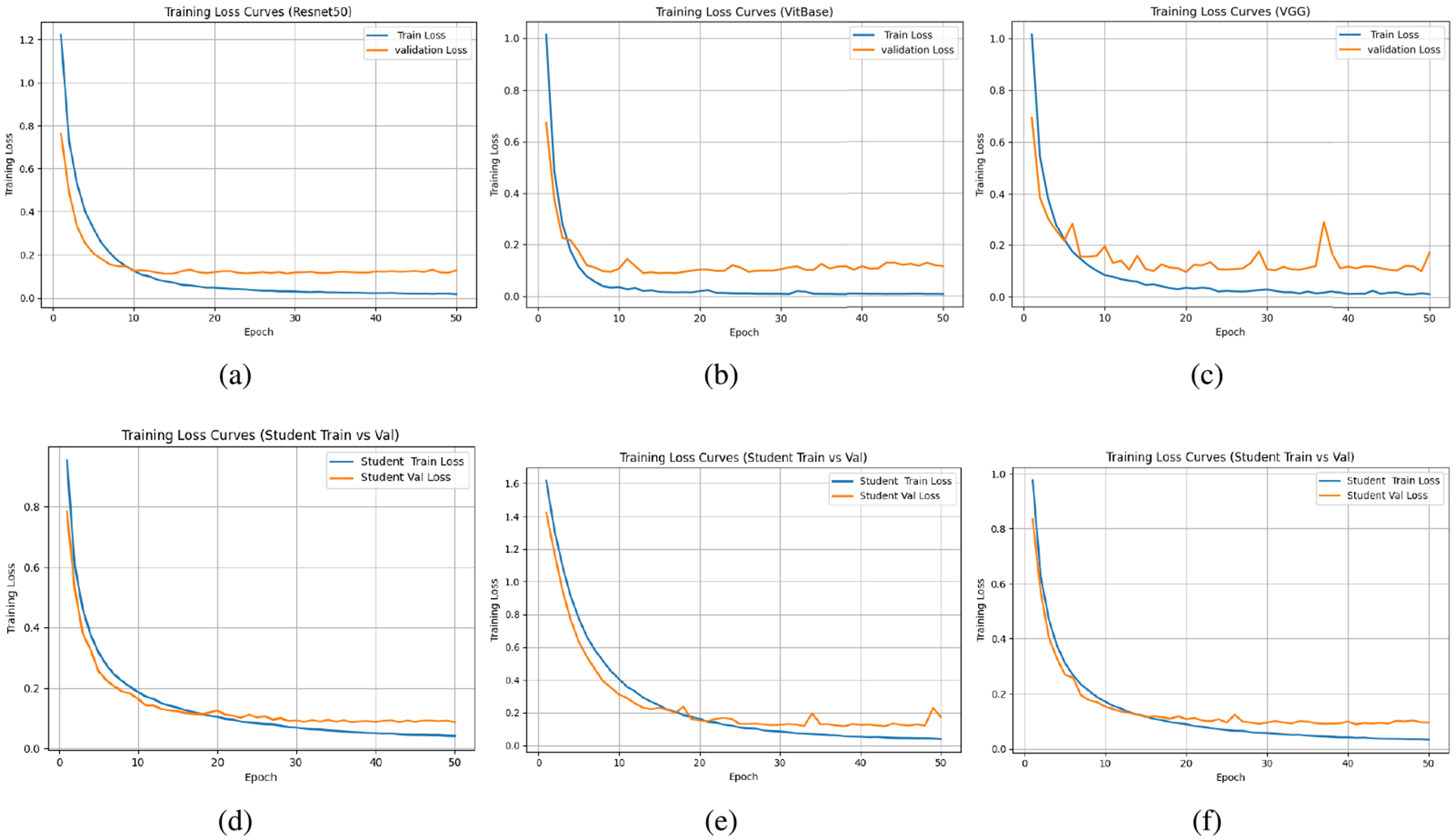
Multiclass image-wise training and validation losses of (a) ResNet50, (b) ViT-Base (c) VGG19 (d) distilled ViT-Base using ResNet50 (e) ResNet152 (f) distilled ViT-Base using ResNet152.

Table 4 and Table 5 show image-wise and patient-wise experimental results respectively. For multiclass image-wise and patient-wise classification, the attention-based architectures ViT-Base, ViT-Large, Swin-Base, and Swin-Large achieved classification accuracies of 91.66%, 92.43%, 88.22%, and 90.12%, respectively, under the image-wise split, and 88.40%, 90.23%, 86.92%, and 88.66% under the patient-wise split. In comparison, CNN models, including ResNet-50, ResNet-152, VGG19, and EfficientNet, obtained accuracies of 89.51%, 90.30%, 87.42%, and 90.42% for image-wise evaluation, and 86.50%, 87.30%, 85.42%, and 87.40% for patient-wise evaluation. The top patient-wise accuracy of 96.87% is obtained using a ViT-Large student with a ResNet152 teacher. We note that all reported results correspond to the mean performance over multiple independent runs with different random seeds. The superior performance of attention based models can be attributed to their global receptive field, which enables the modeling of long-range dependencies across the entire image, unlike convolutional networks which rely on local receptive fields and hierarchical feature aggregation, transformer-based architectures capture global contextual relationships from early layers, allowing spatial information to be integrated more effectively. ViT achieves comparable accuracy to the ResNet baseline in fewer training epochs, as shown in Fig 8. When KD is applied, convergence requires more epochs; however, the distilled models ultimately achieve higher performance, as illustrated in Fig 8. This property is particularly advantageous for histopathological images, where discriminative factors are often characterized by subtle morphological variations and fine grained textural differences rather than coarse structural cues. Attention based models are therefore inherently more expressive in capturing these microscopic details.

When using a patient-wise split, all images from the same patient are strictly assigned to a single subset (training, validation, or testing). Under this split, classification accuracies are consistently lower than those obtained with image-wise evaluation. This decrease is expected and primarily reflects the elimination of data leakage that can occur in image-wise splits. In the image-wise setting, image patches extracted from the same histopathological images may appear in both training and evaluation sets. Since such patches share highly similar morphological patterns, staining characteristics, and acquisition conditions, the model is exposed during training to visual features that also appear during evaluation. This leads to an optimistic bias in performance estimates. In contrast, the patient wise protocol enforces strict independence between training and evaluation data. The model must generalise across patients with varying tissue preparation, staining variability, and morphological heterogeneity. Consequently, the resulting performance provides a more realistic and clinically relevant assessment of the model’s generalization capability. Importantly, despite the overall reduction in accuracy, the relative performance trends across models remain consistent between the two evaluation strategies. This indicates that the superior performance of attention based architectures is not an artifact of data leakage, but rather reflects their enhanced capacity to capture global contextual information and fine grained structural patterns.

We further compare our approach with several state of the art feature and logit based distillation methods under both patient wise and image-wise evaluation protocols. Under the image-wise split, DeiT Base achieved an accuracy of 92.50% by incorporating an additional learnable distillation token. FitNet attained 92.03% by enforcing feature regression between intermediate layers of the teacher and student models. CSKD-S and CSKD-B achieved accuracies of 92.32% and 94.22%, respectively. TAT obtained 91.54% by transferring attention maps between the teacher and student networks. while SemCKD reached 93% through cross layer feature alignment between heterogeneous architectures.

For the patient wise evaluation, DeiT Base achieved an accuracy of 90.42%, whereas FitNet obtained 89.49%. CSKD-S and CSKD-B achieved accuracies of 90.28% and 92.33%, respectively, TAT achieved 90.54%, Cross Distillation achieved 91.20% by aligning representations across multiple layers. Despite their strong performance, a common limitation shared by most of these distillation based approaches lies in the arbitrary or heuristic selection of layer correspondences between the teacher and student models. In many cases, layer alignment is either fixed a priori or chosen at random, without explicitly accounting for representational compatibility between layers. Such arbitrary alignment can introduce negative transfer when knowledge is distilled between layers that encode features at substantially different representational levels. This mismatch is particularly pronounced in heterogeneous settings, such as CNN-Transformer distillation, where architectural inductive biases differ significantly. As a result, distilling knowledge between misaligned layers may constrain the student’s learning process and limit its achievable performance. In contrast, our approach explicitly addresses this limitation by data driven layer alignment, ensuring that knowledge transfer occurs only between layers exhibiting high representational similarity. By systematically identifying compatible layer pairs prior to distillation, our method mitigates negative transfer and enables more effective knowledge propagation across architectures, leading to improved and more stable performance under both evaluation protocols.

For clarity, we first summarize the annotations used in the experimental tables. R denotes response-based distillation only, where the student mimics the output probability distribution of the teacher. F refers to feature-based distillation only. CKA indicates that Centered Kernel Alignment is used exclusively as the criterion for layer matching between teacher and student. R + CKA + KCCA represents the full proposed framework, which combines response based distillation with CKA and KCCA guided layer alignment. Furthermore, Early and Late denote distillation applied only to early stage or late stage layers of the network, respectively. We evaluated multiple combinations of convolutional and attention based teacher student architectures under different distillation strategies, including response based only (R), feature based only (F), CKA based layer alignment, and the full combination of response based distillation with CKA and KCCA. In addition, we examined the impact of distilling knowledge from different network stages (early versus late layers) to assess the contribution of each stage to the overall performance.

Using response based distillation only, the ResNet-50 → ViT-Base configuration achieved classification accuracies of 91.89% for the image-wise split and 88.59% for the patient wise split. While these results are relatively strong, response based distillation primarily transfers knowledge at the output level. Consequently, the supervision signal received by the early and intermediate layers of the student is limited, as the student is mainly encouraged to mimic the teacher’s output probability distribution rather than its internal representations. When using feature based distillation only with first and last layer supervision, the performance improved to 92.44% (image-wise) and 89.84% (patient-wise). This improvement can be attributed to the richer training signal provided by intermediate representations. However, the absence of an explicit and principled layer alignment strategy may lead to suboptimal knowledge transfer, as features from mismatched abstraction levels can introduce noisy or conflicting supervisory signals. Employing CKA as the sole criterion for layer matching further improved performance to 94.69% for image-wise evaluation and 92.37% for patient-wise evaluation. This gain highlights the importance of representation-level alignment, as CKA enables the identification of layers with similar representational structures without forcing the student to replicate the exact feature geometry of the teacher. As a result, knowledge is transferred in a more flexible and semantically consistent manner, facilitating improved signal flow across the network.

Finally, the full combination of response based distillation with CKA and KCCA guided layer alignment achieved the best performance, reaching 97.31% image-wise accuracy when using ResNet-50 as the teacher. When VGG19 was used as the teacher, the proposed method achieved 95.15%, still outperforming most state-of-the-art KD-based methods by a margin of at least 4% accuracy, as reported in Table 4 and Table 5 for image-wise and patient-wise results. The slightly lower performance observed with VGG19 as a teacher can be attributed to the weaker representational capacity of the teacher itself. In such cases, negative or less informative supervisory signals may limit the effectiveness of knowledge transfer, particularly when compared to stronger teachers such as ResNet50.

Overall, these results demonstrate the effectiveness of selectively distilling knowledge from the most compatible teacher-student layer pairs while preserving the representational freedom of the student. By avoiding arbitrary layer alignment and leveraging representation similarity, the proposed approach enables more efficient and robust knowledge transfer, leading to substantial performance gains across both evaluation protocols. When full weighting is assigned exclusively to late layer distillation, the classification performance degrades, reaching 93.20% for the image-wise setting and 90.12% for the patient wise setting in the ViT-Base × ResNet50 configuration. This decline can be attributed to excessive constraints imposed on the student model at deeper layers, where class specific and highly abstract representations are formed. At these stages, the student is expected to develop task adaptive and architecture specific features.

A distillation signal that is too strong in the later layers risks limiting the expressivity of the student by forcing it to closely mimic CNN specific representations. In this scenario, the CNN teacher may suppress the ViT’s ability to construct its own representation space, effectively transferring inductive biases that are well suited for convolutional architectures but suboptimal for transformer based models. Consequently, the ViT may overfit to local, convolution oriented patterns, thereby reducing its capacity for global reasoning, which is a key advantage of attention based architectures. In contrast, assigning full weighting to early layer distillation yields significantly better performance, achieving 95.26% image-wise accuracy and 94.30% patient wise accuracy. This improvement highlights the role of early layers in learning low level visual primitives such as edges, textures, and basic morphological structures, which are largely shared across architectures. At this stage, distillation acts as a form of regularization that stabilizes training without constraining higher level representational flexibility.

To further contextualize the effectiveness of the proposed method, Table 6 presents a comparison with recent state of the art approaches on the BreakHis dataset, including both CNN based and transformer based models. We note that patient-wise results are not reported in most existing methods. Traditional architectures such as ResNet50 and attention based CNN variants achieve competitive performance, with accuracies ranging from 91% to 92%. More recent approaches leveraging multi scale feature fusion and ensemble learning strategies further improve performance, with the best reported accuracy reaching 98.21% using a Vision Transformer based ensemble model. In comparison, the proposed CKA+KCCA guided knowledge distillation framework achieves an accuracy of 98.82%, outperforming all listed methods while also maintaining strong precision (0.9811) and recall (0.9887). This improvement can be attributed to the effective alignment of intermediate representations between teacher and student models, thanks to the complementary use of CKA and KCCA. Unlike prior methods that rely solely on architectural design or ensembling, our approach enhances the student model’s representation learning capability through cross architecture knowledge transfer, leading to superior generalization performance on histopathological images.

### C. CENTERED KERNEL ALIGNMENT RESULTS

For a batch of 240 images, we partition both the teacher and student networks into three stages (early, middle, and late) based on a self CKA analysis of the teacher. This stage wise decomposition enables finer grained similarity analysis while remaining computationally feasible, allowing more samples per comparison and yielding more stable CKA estimates under memory constraints. For transformer based models, CKA is computed using the CLS token representation, which empirically provides similarity patterns comparable to those obtained using all patch tokens while substantially reducing computational cost. This efficiency enables larger batch sizes, which are essential for robust representational similarity estimation. High CKA similarity between two layers indicates that they induce similar geometric organization over the same set of inputs, reflecting shared factors of variation, whereas low similarity suggests reliance on different discriminative cues. Aligning layers with low compatibility may introduce conflicting gradients and degrade student performance. Therefore, within each stage, we select the top k teacher student layer pairs with the highest CKA scores as candidates for knowledge transfer, ensuring that distillation is restricted to representationally compatible layers. We employ linear CKA due to its computational efficiency and robustness for high dimensional representations. After identifying candidate pairs using linear CKA, we further refine the selection using KCCA. For each candidate pair, KCCA is applied to the corresponding activation matrices, and the mean canonical correlation over the leading components is used as a scalar compatibility score. The pair maximizing this score is selected within each stage.

**FIGURE 9.**
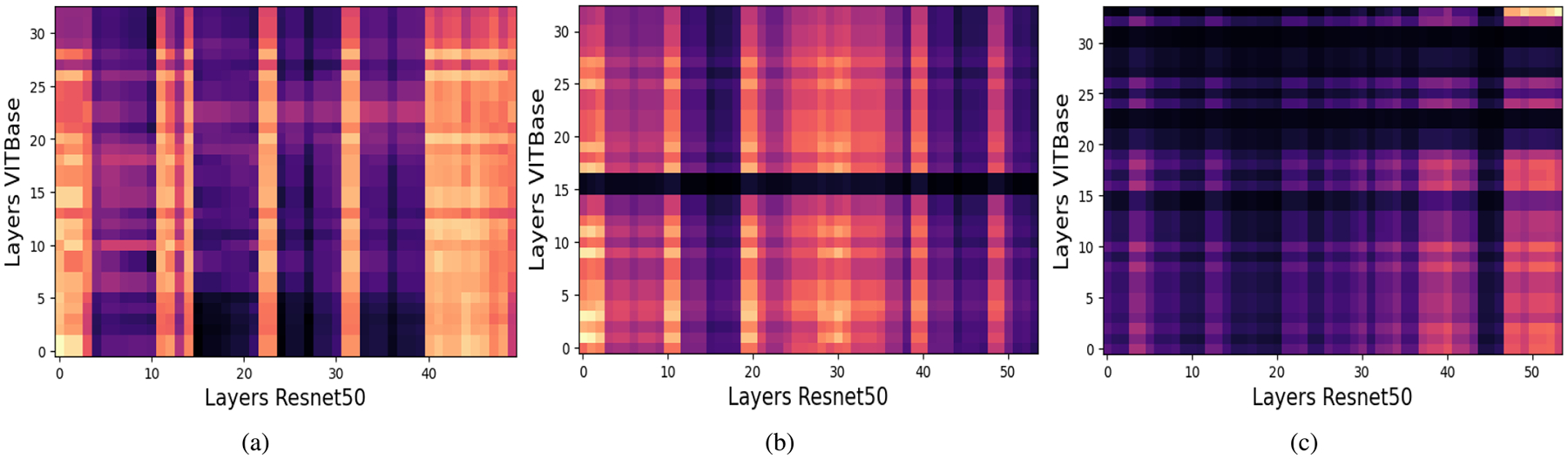
CKA results of ViT-Base x Resnet50 on BreakHis multiclass image-wise (MIM) (a) stage1 (b) stage2 (c) stage3.

**FIGURE 10.**
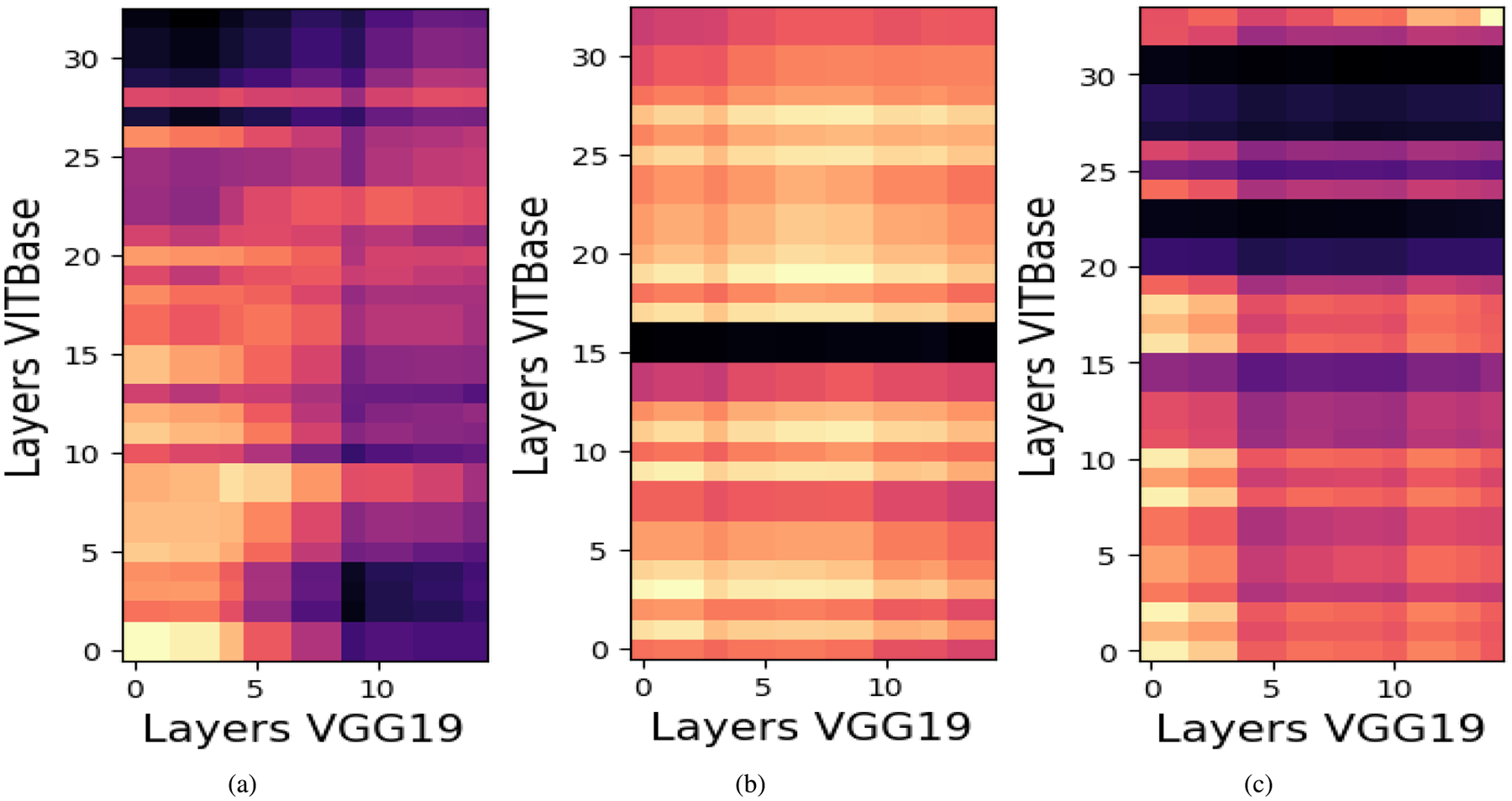
CKA results of ViT-Base x VGG19 on BreakHis multiclass image-wise (MIM) (a) stage1 (b) stage2 (c) stage3.

**FIGURE 11.**
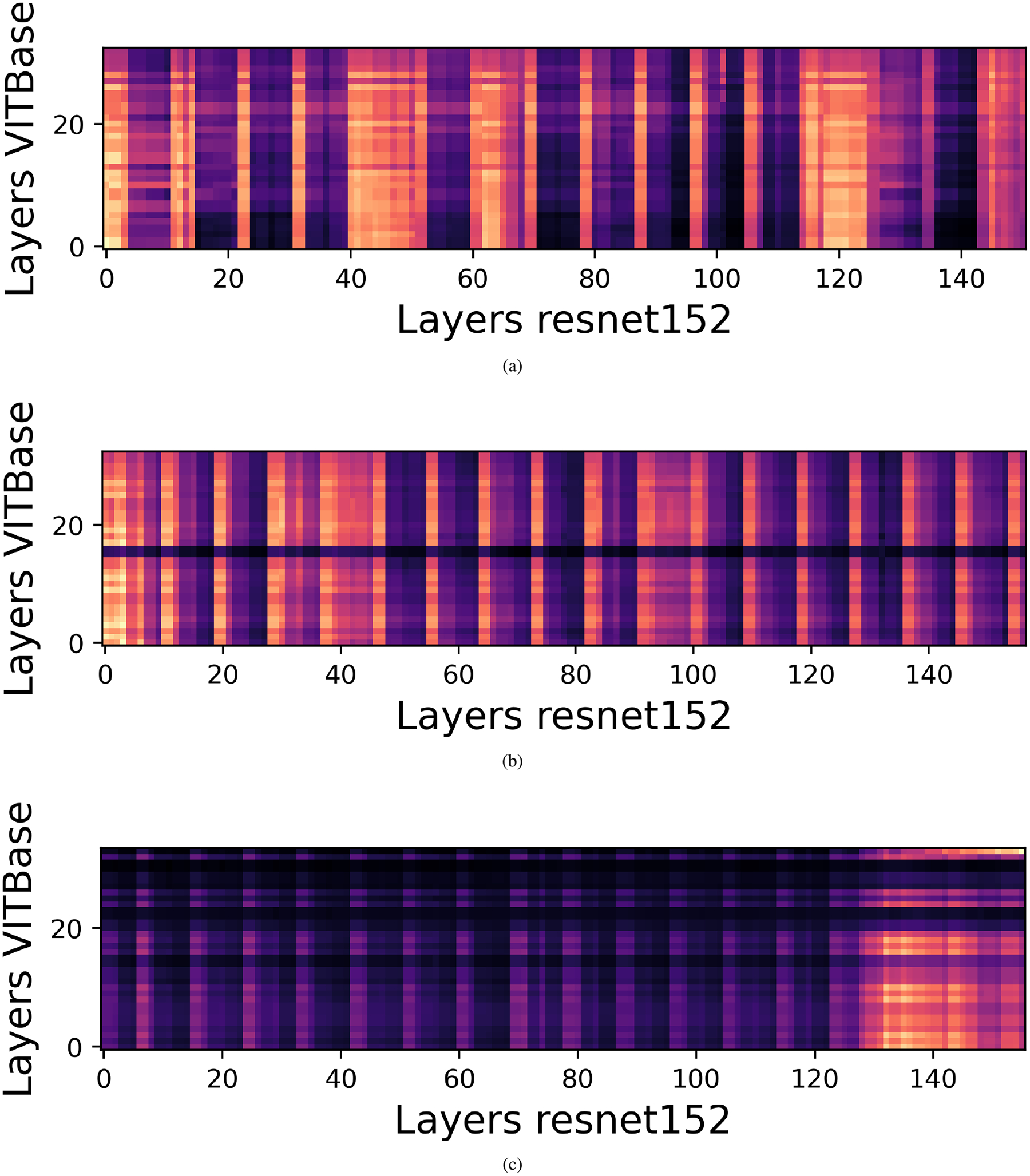
CKA results of ViT-Base x Resnet152 on BreakHis multiclass image-wise (MIM) (a) stage1 (b) stage2 (c) stage3.

Figures 9, Figure 10, and Figure 11 present the resulting CKA similarity matrices for ViT-Base with ResNet50, VGG19, and ResNet152 respectively. In the first stage (Figure 11(a)), broad regions of high similarity are observed, reflecting shared low level features such as edges, colors, and simple textures, which are well suited for feature level distillation. In the intermediate stage (Figure 11(b)), similarity becomes more localized, indicating partial alignment of mid level abstractions despite architectural differences between CNNs and ViTs. This observation is consistent with prior findings by Dorszewski et al [56], who showed that ViTs progressively encode higher level semantic concepts across layers. In the final stage (Figure 11(c)), similarity is sparse and confined to very few layer pairs, reflecting the emergence of highly task specific representations, where indiscriminate alignment may be harmful.

### D. COMPUTATIONAL COST

Table 7 reports the computational cost associated with each alignment strategy. The baseline KD only setting requires 4.77 minutes per epoch with a peak GPU memory usage of 3.44 GB. Incorporating CKA increases the training time to 6.23 minutes per epoch and memory usage to 5.26 GB, reflecting the additional cost of feature similarity computation. When KCCA is further introduced, the training time slightly increases to 6.54 minutes per epoch, with a peak memory usage of 5.87 GB. Overall, while both CKA and KCCA introduce additional computational overhead, the increase remains moderate. In particular, the incremental cost of adding KCCA on top of CKA is relatively small compared to the performance gains achieved. Moreover, since KCCA is computed only once after the warm-up phase and does not affect inference, the proposed method maintains practical efficiency while significantly improving classification performance.

### E. ATTENTION MAPS VISUALIZATION

**FIGURE 12.**
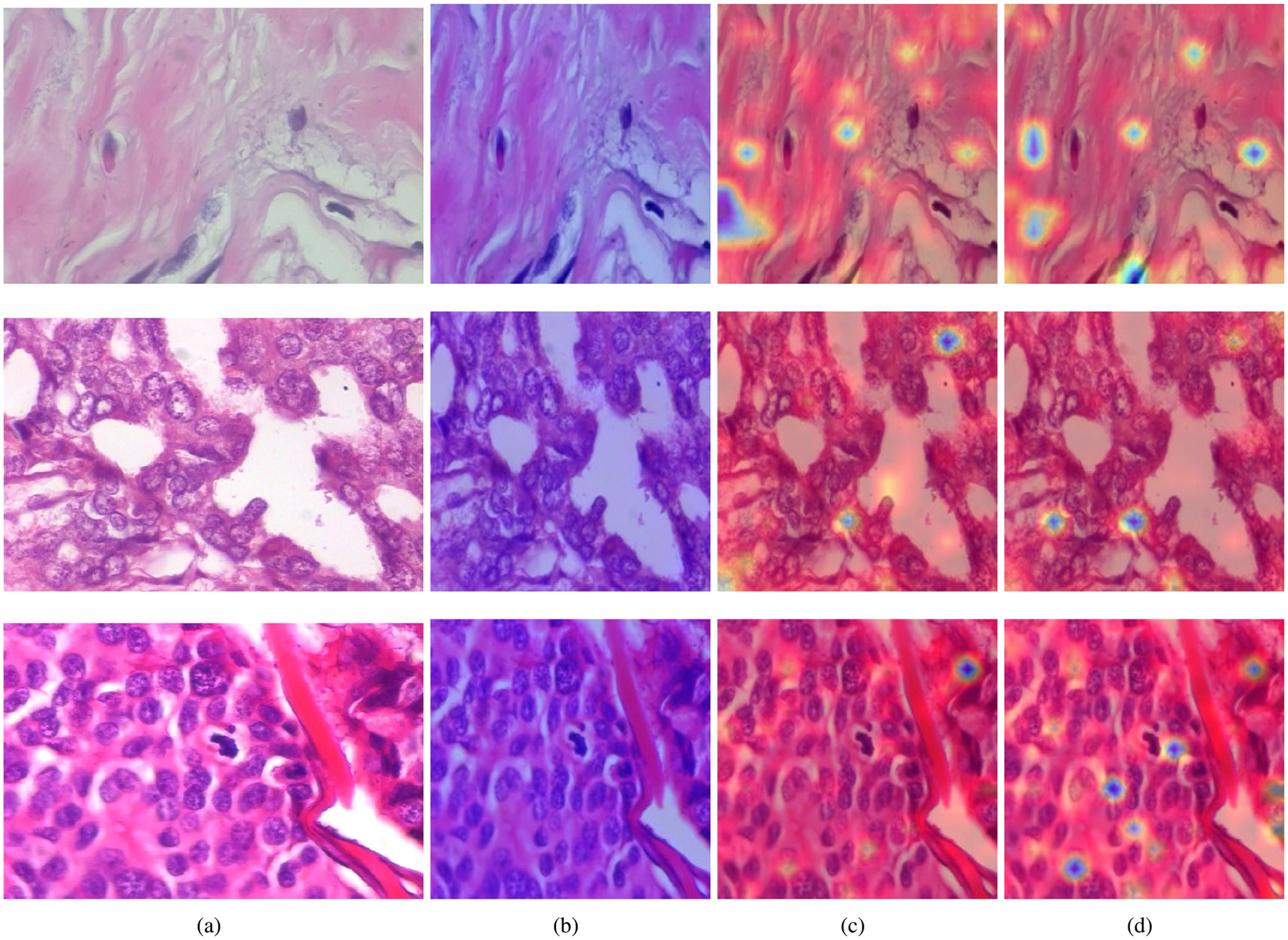
Attention map visualizations comparing four model settings. (a) Original image, (b) untrained model, (c) DeiT baseline, and the (d) proposed method.

Attention maps are visualized using the final transformer block of the ViT. We extract the attention weight tensor of shape H×N×N, where H is the number of attention heads and N the number of tokens. For each head, we retain the row corresponding to the CLS token, discard the CLS to CLS entry, and reshape the remaining N-1 values into a 14×14 spatial attention map, corresponding to the image patch grid. Figure 12 shows the final visualization obtained by averaging the attention maps across all 12 heads, normalizing, upsampling to the original image resolution, and overlaying the result on the input image.

Although attention map interpretation in histopathological images is inherently challenging, several qualitative trends can be observed. The attention maps are presented to illustrate qualitative patterns rather than to support definitive claims about diagnostically relevant regions. The untrained model exhibits diffuse and low magnitude attention across the image, reflecting the absence of learned discriminative behavior. In contrast, both the DeiT baseline (c) and the proposed method (d) display more localized and structured attention patterns, with responses concentrated on specific tissue regions, suggesting that the models attend to potentially diagnostically relevant areas. All visualizations correspond to the mean attention across heads of the final transformer block. Notably, the proposed method often reveals additional or more refined regions of focus compared to DeiT, where supervision is restricted to response based distillation. These supplementary attention regions suggest that incorporating intermediate feature level distillation enables the student to inherit richer structural cues from the teacher’s intermediate representations. Overall, while attention maps should not be interpreted as definitive explanations, the observed differences qualitatively support the claim that the proposed distillation strategy encourages the model to attend to multiple relevant regions rather than relying on a single dominant focus.

## V. FUTURE WORK

While CKA and KCCA provide an effective mechanism for teacher student layer alignment, they remain heuristic tools for optimization rather than definitive measures of semantic correspondence. A more principled understanding of abstraction levels across heterogeneous architectures remains an open research problem, and future work could explore more precise strategies for characterizing and matching representational semantics between teacher and student networks. Similarly, spatial misalignment between CNN and ViT representations continues to pose challenges for feature level distillation. Although the proposed method achieves strong performance, it introduces additional architectural components, requires warm up epochs, and increases optimization complexity. Alternative approaches, such as representation level distillation that directly aligns teacher and student representations using similarity objectives, may offer a more lightweight and architecture agnostic solution. Investigating such strategies could further improve distillation efficiency and generalization across heterogeneous models. Further, testing the proposed layer alignment strategy to check if it can potentially generalizes to other tissue types, organs, cancer classifications, or histopathology tasks is an important research direction.

## VI. CONCLUSION

In this work, we studied knowledge distillation between convolutional neural networks and Vision Transformers in the context of histopathological image analysis. We analyzed how representations evolve across heterogeneous architectures and showed that naive layer or feature matching is often suboptimal due to geometric and semantic mismatches. To address this, we introduced a stage wise layer alignment strategy based on CKA, refined with KCCA, enabling more reliable teacher student matching. In addition, we proposed a feature level distillation mechanism using dedicated distillation tokens to bridge spatial and representational gaps between CNN and ViT features. Experimental results demonstrate that the proposed approach improves ViT performance, particularly in data limited settings, and leads to more structured and informative attention patterns. Overall, this study highlights the importance of representation aware alignment for effective distillation and provides insights into how ViTs can better leverage convolutional inductive biases in medical imaging tasks.

## Figures and Tables

**TABLE 1.** Overview of the BreakHis breast histopathology dataset.

Property	Value
Total images	7,909
Number of patients	82
Benign images	2,480
Malignant images	5,429
Magnifications	40×, 100×, 200×, 400×
Image modality	H&E-stained histopathology

**TABLE 2.** Class hierarchy of the BreakHis dataset.

Superclass	Subclass
Benign	Adenosis (A)
	Fibroadenoma (F)
	Phyllodes Tumor (PT)
	Tubular Adenoma (TA)
Malignant	Ductal Carcinoma (DC)
	Lobular Carcinoma (LC)
	Mucinous Carcinoma (MC)
	Papillary Carcinoma (PC)

**TABLE 3.** CNNs, and ViT models tested in our KD framework.

Model	Architec	Number of Layers	Input Size	Parameters	FLOPs
ViT-Base	Transformer	12	224×224×3	86M	18.33 GFLOPs
ViT-Large	Transformer	24	224×224×3	307M	63.59 GFLOPs
Swin-Small	Transformer	18	224×224×3	50 M	6.01 GFLOPs
ResNet-50	CNN	50	224×224×3	25.6M	4.13 GFLOPs
ResNet-152	CNN	152	224×224×3	60.2M	11.3 GFLOPs
VGG19	CNN	19	224×224×3	143.7M	19.6 GFLOPs
AlexNet	CNN	8	227×227×3	61M	1.5 GFLOPs

**TABLE 4.** BreakHis - Magnification independent multi-category (MIM) - image-wise.

Model	Param	Acc(BreakHis)	Loss	Recall	Precision
ViT-Base	86M	91.66%	0.21	0.9118	0.9139
ViT-Large	304M	92.43 %	0.19	0.9134	0.9226
Swin-Base	88M	88.22 %	0.33	0.8948	0.8781
Swin-Large	197M	90.12 %	0.26	0.9182	0.8995
ResNet50	25M	89.51%	0.28	0.8851	0.9012
ResNet152	60M	90.30%	0.234	0.9256	0.8989
VGG19	144M	87.42%	0.258	0.8790	0.8614
EfficientNet	66M	90.42%	0.239	0.9057	0.8873
**KD Models**					
DeiT-Base	/	92.5%	0.19	0.9094	0.9382
FitNet	/	92.03%	0.23	0.9030	0.9201
CSKD-S	/	92.32%	0.182	0.9190	0.9390
CSKD-B	/	94.22%	0.164	0.9301	0.9523
TAT	/	92.54%	0.203	0.9055	0.8946
SemCKD	/	93.00%	0.172	0.9256	0.9134
**OURS**					
ResNet50 + Vit-Base (R)	111M	91.89%	0.204	0.9022	0.9142
ResNet50 + Vit-Base (F)	111M	92.44%	0.197	0.9020	0.9204
ResNet50 + Vit-Base (R+CKA)	111M	94.69%	0.177	0.9501	0.9140
ResNet50 + Vit-Base (CKA + KCCA)	111M	97.31%	0.113	0.9803	0.9855
ResNet50 + Vit-Base (CKA + KCCA (E))	111M	95.26%	0.164	0.9414	0.9356
ResNet50 + Vit-Base (CKA + KCCA (L))	111M	93.20%	0.214	0.9032	0.9125
VGG19+ViT-Base (R)	230M	92.18%	0.203	0.9223	0.9165
VGG19+Vit-Base (F)	230M	92.76%	0.198	0.9290	0.9105
VGG19+ViT-Base (R+CKA)	230M	92.88%	0.194	0.9340	0.9286
VGG19+ViT-Base (R+CKA + KCCA)	230M	95.15%	0.1214	0.9690	0.9521
VGG19+ViT-Base (R+CKA + KCCA (E))	230M	93.48%	0.1425	0.9242	0.9121
VGG19+ViT-Base (R+CKA + KCCA(L))	230M	92.15%	0.1455	0.9113	0.9232
ResNet152+Vit-Base (R+CKA + KCCA)	146M	98.05%	0.096	0.9737	0.9830
EfficientNet+Vit-Base (R+CKA + KCCA)	152M	97.63%	0.101	0.9651	0.9522
ResNet50+Vit-Large (R+CKA + KCCA)	330M	97.79%	0.099	0.9707	0.9624
ResNet152+Vit-Large (R+CKA + KCCA)	364M	98.82%	0.074	0.9887	0.9811
VGG19+Vit-Large (R+CKA + KCCA)	448M	95.94%	0.121	0.9357	0.9448

**TABLE 5.** BreakHis - Magnification-Independent Multi-category (MIM) classifications - patient-wise.

Model	Param	Acc(Mean)	Loss	Recall	Precision
ViT-Base	86M	88.40%	0.284	0.8730	0.8837
ViT-Large	304M	90.23%	0.263	0.9134	0.8926
Swin-Base	88M	86.92%	0.341	0.8948	0.8781
Swin-Large	197M	88.66%	0.315	0.9182	0.8995
ResNet50	25M	86.50%	0.329	0.8820	0.8679
ResNet152	60M	87.30%	0.322	0.8856	0.8989
VGG19	144M	85.42%	0.356	0.8690	0.8214
EfficientNet	66M	87.40%	0.363	0.8857	0.8773
**KD Models**					
DeiT-Base	/	89.49%	0.219	0.8855	0.8936
FitNet	/	90.42%	0.202	0.9090	0.8789
CSKD-S	/	90.28%	0.198	0.9044	0.8864
CSKD-B	/	92.33%	0.178	0.9273	0.9301
TAT	/	90.54%	0.214	0.8901	0.922
SemCKD	/	91.20%	0.199	0.8865	0.8984
**OURS**					
ResNet50 + Vit-Base (R)	111M	88.59 ± 0.45%	0.284 ± 0.02	0.8633 ± 0.02	0.8955 ± 0.02
ResNet50 + Vit-Base (F)	111M	89.84 ± 0.33%	0.277 ± 0.02	0.9022 ± 0.02	0.8894 ± 0.02
ResNet50 + Vit-Base (CKA)	111M	92.37 ± 0.25%	0.186 ± 0.01	0.9012 ± 0.01	0.9212 ± 0.01
ResNet50 + Vit-Base (CKA + KCCA)	111M	95.87 ± 0.20%	0.124 ± 0.03	0.9456 ± 0.015	0.9523 ± 0.01
ResNet50 + Vit-Base (CKA + KCCA (E))	111M	94.30 ± 0.22%	0.146 ± 0.01	0.9431 ± 0.01	0.9323 ± 0.01
ResNet50 + Vit-Base (CKA + KCCA (L))	111M	90.12 ± 0.32%	0.183 ± 0.01	0.9142 ± 0.02	0.9080 ± 0.02
VGG19+ViT-Base (R)	230M	88.45 ± 0.36%	0.282 ± 0.02	0.8798 ± 0.02	0.8881 ± 0.02
VGG19+ViT-Base (F)	230M	88.98 ± 0.34%	0.276 ± 0.02	0.894 ± 0.02	0.8693 ± 0.02
VGG19+ViT-Base (R+CKA)	230M	89.13 ± 0.33%	0.264 ± 0.02	0.8912 ± 0.02	0.9002 ± 0.02
VGG19+ViT-Base (R+CKA + KCCA)	230M	93.32 ± 0.24%	0.152 ± 0.01	0.9357 ± 0.01	0.9251 ± 0.01
VGG19+ViT-Base (R +CKA + KCCA (E))	230M	90.88 ± 0.30%	0.235 ± 0.02	0.8831 ± 0.02	0.8922 ± 0.02
VGG19+ViT-Base (R +CKA + KCCA (L))	230M	89.15 ± 0.33%	0.248 ± 0.02	0.8753 ± 0.02	0.8611 ± 0.021
ResNet152+ViT-Base (R+CKA + KCCA)	146M	96.05 ± 0.18%	0.122 ± 0.008	0.9527 ± 0.01	0.9648 ± 0.012
EfficientNet+ViT-Base (R+CKA + KCCA)	152M	96.02 ± 0.18%	0.123 ± 0.006	0.9494 ± 0.04	0.9412 ± 0.022
ResNet50+ViT-Large (R+CKA + KCCA)	330M	95.93 ± 0.19%	0.127 ± 0.008	0.9577 ± 0.025	0.9584 ± 0.015
ResNet152+ViT-Large (R+CKA + KCCA)	364M	96.87 ± 0.15%	0.092 ± 0.005	0.9622 ± 0.02	0.9613 ± 0.01
VGG19+ViT-Large (R+CKA + KCCA)	448M	94.28 ± 0.22%	0.142 ± 0.01	0.9401 ± 0.01	0.9249 ± 0.012

**TABLE 6.** Comparison with state-of-the-art methods on the BreakHis dataset.

Ref.	Method	Backbone / Model	Accuracy (%)	Precision	Recall
[49]	Attention-based Deep Learning (ECSA-Net)	CNN-based	91.30	–	–
[50]	Deep Residual Learning	ResNet-50	92.42	–	–
[51]	Multi-scale Feature Fusion Network (BCMNet)	CNN-based	92.65	–	–
[52]	Ensemble Deep Learning	Vision Transformer	98.21	89.84	89.97
**Ours**	**CKA+KCCA Guided Knowledge Distillation**	CNN → ViT	**98.82**	**0.9811**	**0.9887**

**TABLE 7.** Computational overhead analysis of different alignment strategies on BreakHis.

Method	Time/Epoch (min)	Peak Memory (GB)
KD only	4.77	3.436
KD + CKA	6.23	5.255
KD + CKA + KCCA	6.54	5.874
